# A Comprehensive Review on Engineering Release at the Molecular Level: Role of Functional Groups in Polysaccharide-Based Drug Delivery

**DOI:** 10.3390/ma19163430

**Published:** 2026-08-13

**Authors:** Daniel Nicolae Crisan, Teodor Adrian Enache

**Affiliations:** National Institute of Materials Physics, 405A Atomistilor Str., 077125 Magurele, Romania; daniel.crisan@infim.ro

**Keywords:** polysaccharide functionalization, stimuli-responsive targeted drug delivery (SRTDD), dynamic covalent chemistry, controlled drug release, smart biomaterials

## Abstract

Stimuli-responsive drug-delivery systems (SRDDS) have transformed precision medicine by enabling spatiotemporal control over therapeutic release, significantly reducing off-target toxicity while enhancing efficacy at the disease site. Naturally occurring polysaccharides, such as hyaluronic acid, chitosan, alginate, and dextran stand out as premier scaffolds for these “smart” nanoplatforms due to their inherent biocompatibility, biodegradability, and abundance of reactive sites for molecular engineering. This review explores the versatility of polysaccharide functionalization, detailing how the introduction of molecular “switches” allows these biopolymers to sense and respond to specific physiological triggers. We analyze the mechanisms behind acid–labile bonds for pH-triggered release, redox-sensitive bridges for intracellular delivery, and enzyme-cleavable sequences for bio-catalytic activation. By bridging the gap between molecular functionalization and clinical utility, these bio-responsive polysaccharide architectures enable integrated physiological monitoring and theranostic applications. This review highlights the impact of these advancements in overcoming biological barriers, providing a sophisticated blueprint for the next generation of nature-derived, “intelligent” biomaterials in cancer therapy.

## 1. Introduction

Targeted drug delivery (TDD) is a multi-billion industry increasing in size and scope, aimed towards resolving complex healthcare issues. The main objective of TDD is to obtain treatments with higher therapeutic index. The prominent disciplines where TDDs are emerging as state-of-the-art novel therapeutics or promising future solutions, are in oncology, antimicrobial resistance, neurodegenerative disorders, chronic inflammatory and autoimmune diseases, cardiovascular diseases, and gene delivery [1].

In cancer treatments, TDD ensures that the toxic payload is released preferentially at the tumor site, significantly reducing the side effects associated with systemic delivery of chemotherapeutic drugs. The enhanced therapeutic effect by targeting is achieved passively utilizing the enhanced permeability and retention effect of blood vessels in tumors, and actively by functionalizing the drug carriers with ligands that bind to receptors overexpressed at the surface of cancer cells [2]. Furthermore, using nanocarriers protects medication from enzymatic degradation in the body or premature elimination via the kidneys [3]. Additionally, highly potent experimental drugs, that would otherwise be too cytotoxic for systemic delivery, can be encapsulated inside liposomes or polymeric micelles, accumulating at the tumor site and decreasing the exposure to healthy organs [4]. Targeted delivery also addresses the multidrug resistance that tumor cells develop through efflux pumps, by permitting cell penetration through alternative mechanisms (e.g., endocytosis), bypassing these pumps [5].

Many of the advantages of TDD for cancer therapy have direct equivalents in fighting antimicrobial resistance [6]. Improved therapeutic index [7] is achieved by reducing the systemic toxicity of antibiotics, particularly to the kidneys, liver, and healthy gut microbiome, the drug carrier releasing the cargo preferentially at the site of infection [2]. This allows also an increased (10–100 times) local concentration, well above the safe limits of systemic administration, enough to kill moderately resistant bacteria that would otherwise resist a standard dose. In chronic and resistant infections, biofilms produced by bacteria are often the main culprit, as the complex extracellular polymeric substance matrix acts as a physical barrier that slows down or neutralizes the penetration of antibiotics [8]. Charged [9] or enzyme-rich drug carriers [10] can be exploited to physically “drill” through the biofilm and deliver the drug to the bacterial cells. Similar to cancer cells’ efflux pumps, bacteria also have similar mechanisms to spit out antibiotics, or release enzymes (such as β-lactamases) to chemically degrade them [11]. TDD protects and delivers a high dose of the drug and can be combined with efflux pump inhibitors to enhance the drug’s activity. In fact, the main troublemaker of antimicrobial resistance is exposure to sub-lethal doses of active molecules, which TDD addresses by achieving a controlled and sustained release [12].

Neurological and neurodegenerative disorders are a particular subject of TDD. The blood–brain barrier (BBB) is notoriously prohibitive for small-molecule drugs and large-molecule therapeutics alike. Nanocarriers can be functionalized with ligands such as transferrin or insulin that facilitates the transport across the BBB via transcytosis [13], or completely bypassing the BBB by intranasal delivery avoiding the bloodstream completely [14]. Nanocarriers can be used to stabilize fragile cargoes (e.g., mRNA, siRNA, CRISPR-Cas9) that would be enzymatically destroyed in the blood stream before reaching the target sites in the brain, or they can be designed as biodegradable implants that keep a constant dosage over long periods of time, eliminating the requirement for frequent administration [15,16,17]. Furthermore, enhanced nanocarriers can be engineered to release the active cargo in the presence of specific biological triggers, such as high oxidative stress or acidic pH levels characteristic of inflamed neural tissue [18].

In the treatment of chronic inflammatory and autoimmune diseases, the use of TDD presents many of the same advantages as described above, mainly enhancing therapeutic efficacy by increasing the local concentration at the required site while decreasing the free drug in the bloodstream in other parts of the body. Nanocarriers can be engineered to help drugs penetrate difficult to access regions such as the synovial fluid or intestinal lining. Immunosuppressant treatments often present severe side effects (i.e., organ toxicity, increased risk of infections, bone marrow suppression) when circulating freely throughout the body, and encapsulation in a nanoparticle can spare healthy tissue from needless exposure, as well as allowing the use of a lower dose with a similar or better therapeutic effect. In the management of chronic conditions [19], TDD systems can be designed into controlled-release implants that greatly enhance the treatment experience from the patient’s point of view, reducing the frequency of dosing and facilitating the patients’ compliance [20].

In cardiovascular diseases, TDD can be engineered to aid plaque stabilization by delivering anti-inflammatory agents directly to unstable plaque, or to dissolve blood clots using smart particles releasing thrombolytics at the site of the blockage, decreasing risks of bleeding. Since cardiac tissues are hard to penetrate, targeted systems can bypass the endothelial barrier more effectively or deliver cargo to cells directly, which is cornerstone to regenerative medicine and gene silencing therapies [21,22]. Novel cardiovascular drugs, particularly peptide-based or gene therapies, are structurally fragile and can be degraded by enzymes in the blood [23].

As with previous examples, nanocarriers can protect the drugs against enzymatic degradation, and they can be designed for a sustained release, maintaining therapeutic concentrations of the drug for longer periods of time rather than intervals of short spikes and rapid drops. Cardiovascular diseases usually require long-term regimens, so minimal damage to other organs due to drug exposure is particularly vital. As such, nanocarriers coated with ligands that bind to specific heart or blood receptors can be designed to increase the concentration at the target site, while minimizing the exposure to the rest of the body [24].

Gene therapy also greatly benefits from TDD as it addresses the primary hurdle of safe and effective medical treatment (i.e., the delivery problem). In gene delivery, the drug component is usually a fragile strand of RNA or DNA that needs to reach particular cell lines without getting destroyed or causing side effects. The nanocarriers for gene delivery can be engineered to overcome biological barriers such as the BBB, cartilage or cardiac tissue as described previously, and smart features can be incorporated to release the cargo only inside the acidic environment of the target cell’s endosome.

Although the specific design of targeted drug delivery systems varies according to the disease, their therapeutic benefits follow common principles. Table 1 summarizes the major advantages of targeted drug delivery across representative therapeutic areas.

While TDD greatly improves the therapeutic outcome compared to traditional therapies, it is not without inherent flaws. The first generation of TDDs relies on passive or ligand-mediated delivery. This means that the cargo can either leak prematurely before reaching the target site, causing systemic side-effects, or the cargo can be protected from leaking and degradation too strongly, to the extent that the release can be incomplete for desired therapeutic results. The logical evolution to these systems was the stimuli-responsive targeted drug deliveries (SRTDD), also referred to as “smart” or “triggered” delivery.

These subtypes of TDDs are engineered to protect and lock the cargo inside the carrier, until a physical or chemical change causes the destabilization of the nanoparticle carrier or release of drug molecules from labile chemical bonds. As a consequence, this allows the localized delivery of high concentrations of drugs, the fine control of release kinetics and decreased systemic toxicity. The engineering of SRTDDs is largely dependent on the nature and versatility of the nanocarrier. The materials chosen must be flexible towards multiple types of chemical modifications (ideally orthogonal) to allow introduction of target specific ligands and the stimuli responsive motifs, while being biocompatible and biodegradable. Herein, we explore why polysaccharides are particularly suitable as nanocarriers for SRTDD, and the types of stimuli that can be engineered in these systems.

## 2. Why Polysaccharides

Natural polysaccharides are biopolymers composed of sugar units bound by glycosidic linkages. Relating to the design of nanocarriers, four polysaccharides have been the focus of the most R&D efforts (i.e., chitosan, hyaluronic acid, alginate and dextran), but others are emerging due to their unique properties (e.g., heparin, pectin, cellulose, starch).

Chitosan is a linear, randomly distributed, *β*(1→4) linked, *D*-glucosamine and *N*-acetyl-*D*-glucosamine polymer. At physiological and acidic pH, it is positively charged, making it suitable for binding negatively charged DNA/RNA and interacting with negatively charged cell membranes [25]. It is one of the few naturally occurring cationic polymers that is highly biocompatible and does not elicit a significant inflammatory response or a toxic effect. Structurally, it is similar to the glycosaminoglycans found in the extracellular matrix. Being susceptible to enzymatic hydrolysis, it is biodegradable [26], releasing harmless amino sugars as products, which are excreted or recycled by the body. The primary amine present at the C-2 position can be used for a variety of functional group transformations using click and non-click chemistry [27].

Hyaluronic acid (HA) is a natural component of the human extracellular matrix, so it is inherently biocompatible and does not normally trigger an immune response, except when low molecular weight fragments (10–50 kDa) are detected and interpreted as Damage-Associated Molecular Patterns. Hyaluronic acid has high affinity for CD44 receptors, often overexpressed in cancer cells, and can act as a built-in targeting ligand [28,29]. Structurally, it is a non-sulfated glycosaminoglycan composed of alternating *D*-glucuronic acid and *N*-acetyl-*D*-glucosamine. While the carboxylic acid group at the C-5 position of the *D*-glucuronic acid confers the polymer with anionic character, the lack of sulfate groups results in a lower charge density compared to highly charged polysaccharides like heparin. The absence of sulfate groups allows hyaluronic acid to form specific secondary structures stabilized by hydrogen bonds that can trap large amounts of water. Functionalization of the carboxylic acid group is usually carried out via EDC/NHS activation and reaction with nucleophiles or via various esterification strategies.

Alginate is also a negatively charged linear polysaccharide, with a higher charge density compared to HA. Structurally, it is composed of alternating *β*-*D*-mannuronic acid and *α*-*L*-guluronic acid sugar groups, with carboxylic acid groups present on every sugar group compared to alternating sugar groups in HA. This allows alginate to bind divalent cations like Ca^2+^ and form stable, porous structures that can be exploited [30]. In low pH environments, the alginate macromolecular structures shrink and tighten while in high pH, competitive binding causes the alginate chains to unzip and release cargo. Functionalization can be carried out at the carboxylic acid sites, but that can lead to a decreased ability to form regular and stable pores, although it can be mitigated by carrying out functionalization at the mannuronic sugar unit since cationic ligands bind preferentially at the guluronic unit [31]. Nonetheless, other functionalization reactions can be carried at the hydroxyl sites without affecting this property of alginate.

While the previous three biopolymers were examples of linear and charged structures, dextran is a complex branched *α*-glucan, water soluble and neutral [32]. It has the advantage that it exhibits low protein absorption, preventing the formation of protein corona, which leads to immune tagging and clearance by macrophages. This “stealth” property is achieved due to the steric hindrance caused by the branched structures, and by high hydrophilicity, which creates a hard-to-displace hydration layer. Dextran can be functionalized through its hydroxyl groups [33]. Through etherification and esterification, amphiphilic or charged polymers can be created to bind and trap a variety of types of drug molecules (i.e., lipophilic, charged). Oxidation converts the hydroxyl groups into aldehydes that can form Schiff base bonds with amine-containing molecules. Other modifications with bifunctional molecules can introduce complex stimuli-responsive properties that will be covered later.

The selection of an appropriate polysaccharide backbone represents the first step in designing advanced drug delivery systems, as each polymer provides distinct biological and physicochemical characteristics. However, while the backbone determines properties such as biocompatibility, biodegradability, targeting potential, and processability, the incorporation of specific functional groups ultimately governs the mechanism and precision of controlled drug release. The principal characteristics of the four most widely used polysaccharides in drug delivery are compared in Table 2.

## 3. Types of Stimuli: Endogenous vs. Exogenous

In the context of stimuli-responsive targeted drug delivery, two broad categories of stimuli can be classified: endogenous and exogenous [34,35]. Endogenous stimuli are biochemical cues found within the body, often specific or overexpressed at the target site like a tumor or inflamed tissue. The most commonly exploited endogenous stimuli are pH gradients, redox potential gradients, enzyme expression profiles and glucose concentration gradients. pH gradients are prevalent in the acidic environment of tumors and endosomes, with pH varying between 4.5–6.5 compared to 7.4 in healthy blood. Redox potential gradients are driven by high concentrations of glutathione (GSH) or reactive oxygen species (ROS). These occur primarily transitioning from the extracellular space (2–20 µM GSH) to the cytosol (2–10 mM GSH), triggering rapid cleavage of redox-responsive bonds.

Cancer cells typically exhibit even higher levels (4–10 times) of GSH than healthy cells. Enzyme expression profiles represent the most specific endogenous triggers [36]. Ranging from hydrolases like esterases and lipases to proteases that target specific peptide sequences, including zinc-dependent matrix metalloproteinases (MMPs) in the extracellular matrix and glycosidases that cleave complex sugars, a vast ‘toolbox’ is available to target a variety of sites. Glucose concentration gradients are usually fluctuating, representing a dynamic stimulus rather than an on–off switch. This property can be exploited for chronic metabolic management by engineering systems that respond reversibly to release or suppress the drug (e.g., insulin).

Exogenous stimuli are physical triggers applied from outside the patient’s body to control the release of drug in a localized and time dependent manner [37]. Most of these stimuli take advantage of physicochemical properties of polymeric materials or nanoparticles.

For example, thermo-responsive polymers loaded with drugs undergo phase changes to release the cargo. Depending on the depth of tissue that needs to be penetrated, different heat administration regimens can be applied. During superficial and very shallow (0–10 mm) (e.g., dermal or superficial tumors) thermal stimulation, contact heating, visible light sources or short-range near-infrared (NIR) light can be used. For subcutaneous fat, muscle layers or lymph nodes (1 cm–3 cm), NIR light can be used to excite nanomaterials and create localized heating effects (i.e., photothermal effect). Beyond this depth penetration, light-scattering processes are significant, leading to a loss in energy density, below required efficient triggered drug release. When targeting internal organs or deep-seated tumors found in deep tissues, localized heating is achieved by converging multiple beams of different types of energy to focus on the target site and induce heating in the SRTDD. Focused ultrasound is the most precise since it can achieve exact phase-transition temperatures (40–42 °C) at the target without heating the tissue along the path, while alternating magnetic fields can be used to achieve any depth required without interference from bones, muscles, and fat.

One of the other highly exploited exogenous stimuli, relies on photo-sensitivity of chemical bonds. UV-Visible or NIR light can be used to modify the structure of the carrier via cis-trans isomerization or physically breaking bonds. These can be exploited to open engineered pores, swell a hydrogel, or physically break covalently attached molecules, to release the drugs. Because of poor tissue penetration of UV-Visible and NIR light, photo stimulation can be exploited mostly in superficial and dermal application, or by using invasive methods such as optical fibers through catheter and radial diffusers or optical capsules that can be ingested or implanted.

Magnetic fields can be exploited to physically direct magneto-susceptible nanocarriers to the target sites. Although normally these are metallic nanoparticles, hybrid organic or inorganic materials that incorporate magnetic nanoparticles with biocompatible coatings are becoming a key area of research. Often, magnetic responsiveness is combined with thermal release as magnetic fields can cause localized heating that induces drug release or hyperthermic treatment (42 °C can kill tumor cells while only reversibly damaging healthy cells). Other exogenous SRTDDs use ultrasound or electricity. Deep tissue, non-invasive, ultra-sound responsive delivery systems work by oscillating microbubbles, causing physical shockwaves that deliver drugs into cells. Electro-responsive systems take advantage of conductive polymers that can swell or shrink, functioning as a drug pump, or use redox-active groups to change the hydrophilicity of the carrier.

To translate these endogenous microenvironmental gradients and exogenous physical triggers into controlled therapeutic action, specific functional groups must be incorporated into the polymer scaffold. Section 4 examines the chemical linkages and reactive moieties that serve as molecular switches in stimuli-responsive polysaccharide platforms.

## 4. Functional Group Modifications for SRTDD

### 4.1. pH Gradients Responsiveness

Aside from manipulating pH responsiveness through the backbone by acidic and basic functional groups, which can affect drug release through particle swelling/contracting, or through changes in solubility, there are pH-dependent chemical bonds that can be used to modify the stability of nanocarriers [38]. Hydrazone bonds are reversible bonds formed between hydrazine derivatives and aldehydes/ketones [39,40]. While highly stable at pH 7.4 (i.e., healthy blood), hydrolysis occurs in the more acidic (pH 4.5–6.5) environments of tumors and interior of endosomes and lysosomes [41].

Similarly, acetals and ketals can be formed from the reaction of alcohols with aldehydes/ketones [42], but with increased stability at near-neutral pH compared to hydrazones, making them ideal release triggers for pH values nearer to 5.0 and longer circulation times [43,44]. Furthermore, the alcohol and carbonyl byproducts of the hydrolysis are usually more biocompatible than the hydrazine derivatives. In the context of polysaccharides, the abundance of hydroxyl groups naturally present provides an existing platform for engineering acetal linkages.

Other useful linkages that take advantage of rich hydroxyl environments are boronate esters [45], usually with modified boronic acids that have lower pKa values compared to phenylboronic acid (pKa 8.8) to make them suitable for physiological applications. Orthoesters are another class of useful pH-sensitive group in SRTDD [46]. These bonds generally hydrolyze faster than acetal or hydrazone counterparts and allow zero-order release kinetics, allowing constant drug delivery, useful in long-term implants. Highly particular linkages such as the cis-aconityl bond [46] are efficient acid-labile bonds used for delivery of drugs containing primary amines such as Doxorubicin. The three carboxylic acid groups in cis-aconitic acid serve as an anchor to the drug carrier, an attachment point for the drug via an amide bond, and a free carboxylic acid that acts as an internal self-catalyst under the right pH conditions (i.e., pH inside endosome).

**Figure 1:**
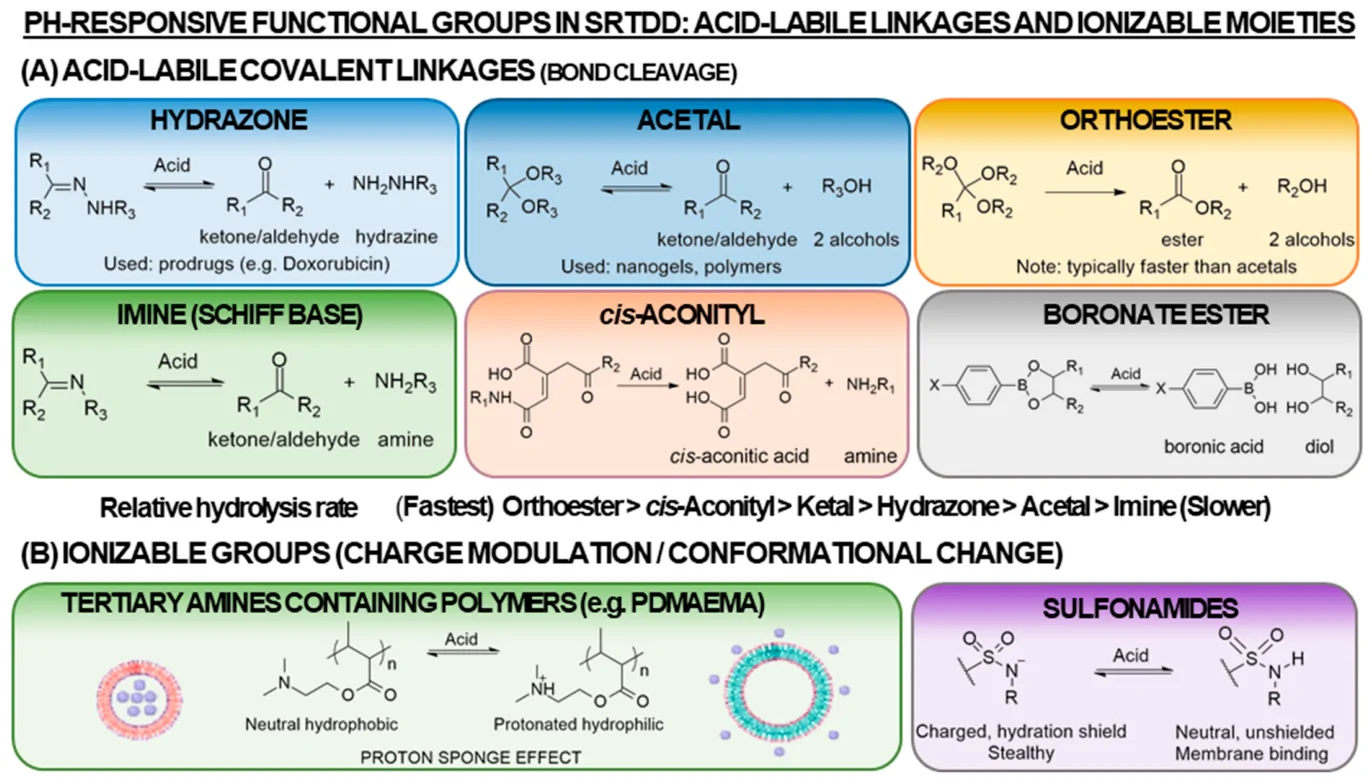
Representative acid-responsive functional groups employed in polysaccharide-based drug delivery systems. (**A**) Acid–labile covalent linkages, including hydrazone, acetal, orthoester, imine (Schiff base), cis-aconityl, and boronate ester functionalities. (**B**) Ionizable functional groups that regulate carrier charge and conformation through reversible protonation–deprotonation, illustrated by tertiary amines and sulfonamides. Google Gemini was used to generate the preliminary visual concepts and visual artwork representations as blue-prints for this figure. Prompts outlining chemical linkages, biological pathways, and structural release mechanisms were provided to guide visual creation. The study conception, data interpretation, and final validation of the visual accuracy were exclusively performed by the authors.

Acid-responsive functional groups constitute one of the most extensively investigated molecular strategies for engineering-controlled drug release because they exploit the pH gradients that naturally exist between healthy tissues and pathological microenvironments. Unlike conventional carriers, whose release depends primarily on passive diffusion, these systems respond to acidification through two complementary molecular mechanisms: acid-labile covalent bond cleavage and reversible ionization. The principal acid-responsive functional groups discussed in this review, together with their corresponding activation mechanisms, are summarized in Figure 1.

Acid-labile-covalent linkages undergo hydrolysis under mildly acidic conditions characteristic of tumor tissues, inflamed sites, endosomes, and lysosomes, leading to carrier degradation or cleavage of polymer–drug conjugates. Among the most widely employed functionalities are hydrazones, acetals, orthoesters, imines (Schiff bases), and cis-aconityl linkages, each exhibiting distinct hydrolysis kinetics that depend on both the chemical structure of the linkage and the surrounding molecular environment.

Boronate esters represent a distinct class of acid-responsive functionalities because their behavior is governed primarily by reversible boronic acid–cis-diol complexation rather than direct hydrolysis. Acidification shifts the equilibrium toward dissociation of the boronate ester, whereas physiological pH favors the formation of stable boronate–diol complexes.

Unlike acid-labile systems, ionizable functional groups regulate drug release without covalent bond cleavage. Instead, reversible protonation and deprotonation modify polymer charge, hydrophilicity, and intermolecular interactions, producing changes in carrier swelling, permeability, and structural organization. Protonation of tertiary amines increases electrostatic repulsion and water uptake, frequently generating the proton sponge effect that promotes endosomal escape and intracellular drug release. Conversely, sulfonamide-containing polymers undergo pH-dependent ionization that regulates surface charge, hydrophobicity, carrier stability, cellular uptake, and drug diffusion. Together, these complementary mechanisms provide precise control over carrier stability, intracellular activation, and therapeutic release.

### 4.2. Redox Potential Gradients Responsiveness

The main functional group responsive to glutathione (GSH) concentration changes is the disulfide bond (-S-S-) [47]. Mechanistically, GSH and disulfide bonds interact through thiol-disulfide exchange in a concentration-dependent manner. In practice, this means disulfides are stable in circulation and labile in the cytosol or cancer cells [48]. Disulfides can be exploited as a linker for prodrugs, as crosslinkers in nanogels and particles (including cysteine-rich peptide-based peptides), or to attach “stealth” coatings that protect the nanocarrier until bond exchange occurs.

More recently, diselenides and ditellurides analogues of disulfides have been explored as alternatives due to their lower bond energy (172 kJ/mol and 126 kJ/mol vs. 240 kJ/mol respectively). This makes them more sensitive to redox changes (i.e., lower concentration of GSH), but it also provides sensitivity to reactive oxygen species (ROS) in a reversible manner [49]. Niche applications, such as targeting hypoxic microenvironments can be achieved with aryl-azo groups which undergo reductive cleavage to form two aromatic amines or nitroaromatic groups which are reduced to hydroxylamine or amine groups affecting the solubility of the nanocarrier [50].

Redox imbalance and hypoxia are hallmarks of numerous pathological conditions, particularly solid tumors, chronic inflammation, and certain bacterial microenvironments. Unlike pH-responsive systems, which rely primarily on extracellular acidification or endosomal maturation, redox- and hypoxia-responsive drug delivery systems exploit disease-specific biochemical signatures, including elevated intracellular GSH, increased ROS, and oxygen-deficient environments.

**Figure 2:**
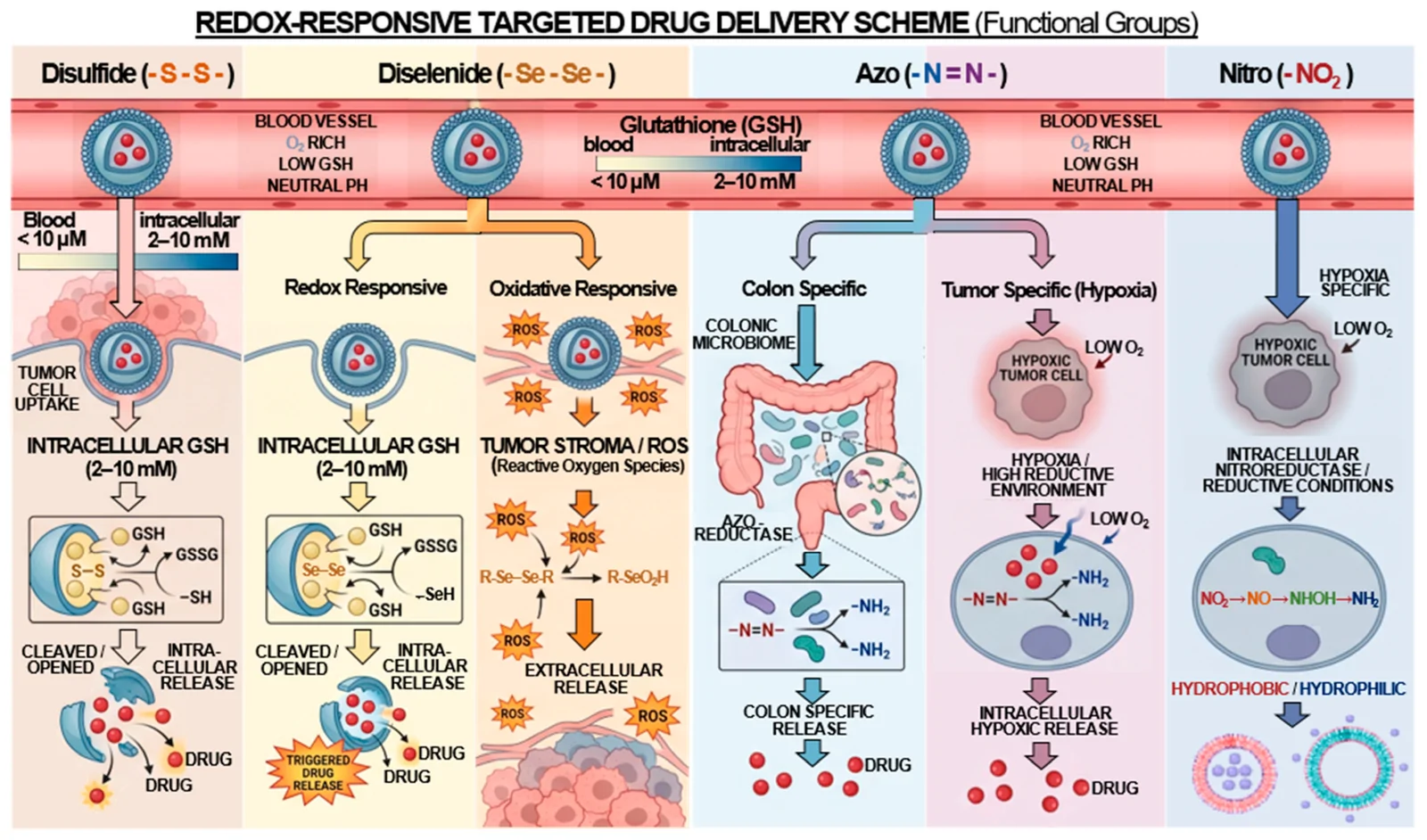
Representative redox- and hypoxia-responsive functional groups employed in polysaccharide-based drug delivery systems. Disulfide, diselenide, azo, and nitro functionalities respond to intracellular reducing environments, oxidative stress, or hypoxia to trigger selective carrier activation and drug release. Google Gemini was used to generate the preliminary visual concepts and visual artwork representations as blue-prints for this figure. Prompts outlining chemical linkages, biological pathways, and structural release mechanisms were provided to guide visual creation. The study conception, data interpretation, and final validation of the visual accuracy were exclusively performed by the authors.

Depending on their chemical structure, responsive functional groups undergo irreversible bond cleavage, reversible redox transformations, or enzymatically catalyzed reduction, resulting in selective carrier activation and controlled intracellular drug release. The principal redox- and hypoxia-responsive functional groups discussed in this review and their corresponding activation mechanisms are summarized in Figure 2.

Among redox-responsive functional groups, disulfide linkages undergo glutathione-mediated thiol–disulfide exchange following cellular internalization, promoting rapid intracellular drug release in GSH-rich environments.

Diselenide bonds possess lower bond dissociation energies than their sulfur analogues and therefore exhibit dual responsiveness toward both intracellular glutathione and reactive oxygen species, enabling activation under reducing as well as oxidative conditions. In contrast, azo groups are activated through enzymatic reduction catalyzed by azoreductases, enabling either colon-specific drug delivery or hypoxia-triggered activation depending on the biological environment.

Similarly, nitro-containing functional groups are reduced by intracellular nitroreductases under oxygen-deficient conditions, generating amino derivatives that destabilize the carrier and promote selective drug release. Together, these complementary molecular mechanisms translate disease-associated redox gradients and hypoxia into highly selective therapeutic activation, thereby improving drug efficacy while minimizing premature extracellular drug leakage and systemic toxicity.

### 4.3. Enzyme Profile Expression

Enzyme responsiveness is considered the most selective targeting strategy in SRTDD. This approach benefits from high substrate specificity, precise pathological localization (i.e., particular enzymes are overexpressed only at the site of diseased tissues) and biocatalytic amplification (i.e., even low concentrations of enzyme can release a large quantity of drugs from a nanocarrier).

The targeted chemical bonds can be quite generic or very specific sequences that only ‘react’ to particular enzymes [49,51]. The main generic bonds targeted are amides, esters, phosphodiester, glycosidic and azo bonds. Amides are cleaved by broad-spectrum proteases and peptidases and are frequently used to deliver drugs in the form of prodrugs covalently attached to the polymeric nanocarrier.

The blood and the liver are especially rich in carboxylesterases and cholinesterase, prime targets for ester substrates. Often, these are exploited in multistep activation sequences of drug delivery where several enzymatic processes need to happen before the active form of the drug is revealed to the target. Phosphate groups are usually employed to improve the solubility of hydrophobic drugs, taking advantage of their high stability in blood. They undergo rapid enzymatic cleaving in areas rich in alkaline phosphatase or acid phosphatase such as inside lysosomes or on cell membranes, releasing the poorly soluble drug at the target site. Glycosidic bonds such as those found in glucuronic acid or galactose can be broken by β-glucuronidase and β-galactosidase expressed in necrotic areas of tumors.

While the previous four examples dealt mostly with bonds broken by human enzymes, azo bonds are broken by azoreductases expressed by microflora. As such, they find a niche application in colon-specific drug delivery, wherein the drug carrier passes intact through the stomach and small intestine but breaks down rapidly in the bacteria-rich large intestine. In the case of bonds that are cleaved by particular enzymes, certain amino acid sequences are introduced either into the overall structure of peptides (often combined with crosslinking groups and charged functional groups that facilitate particle formation), or covalently attached to polymeric nanocarriers. They can serve as pro-drug anchors or as breaking points for nanocarrier destabilization followed by drug release.

Depending on the target enzyme, the following sequences are identified as useful for engineering SRTDD. Sequences such as GFLG, GGFG or Val-Cit (Citrulline) are cleavable by Cathepsin B active primarily in lysosomes and overexpressed in cancer metastasis, arthritis, pancreatitis and cardiovascular diseases [52]. PLGLAG sequences are cleaved between Gly and Leu by Gelatinases MMP-2 and MMP-9 responsible for extracellular matrix degradation (particularly type IV collagen) and overexpressed in tumor metastasis, angiogenesis and inflammatory diseases. Caspase-3, involved in apoptosis-triggered release, cleaves DEVD sequences and can be used to create a self-sustaining cycle that spreads the death signal throughout the targeted issue. AAN sequences are targets for Legumain (asparaginyl endopeptidase), which is overexpressed in most solid tumors and rarely in healthy tissue.

The sequence HSSKLP is highly specific for prostate-specific antigen, allowing the drugs inside the nanocarriers to remain masked everywhere else in the body except for the prostate or prostate tumor sites [48]. The Furin enzyme (PACE or PCSK3) found in the trans-Golgi network, which cleaves RRKR or RVKR sequences, is a target for potential antiviral and anticancer treatment. While the aforementioned enzymes, with their respective amino acid sequences targets, are examples of endogenous enzymes, bacterial enzymes are also useful targets for sequence specific release. For instance, ALAL, GGPLGPR and PLG-L sequences are cleaved by LasB and secreted by *P. aeruginosa*, responsible for several severe opportunistic infections in immune compromised patients [53].

Similarly, peptide bonds on the carboxyl side of glutamic acid or aspartic acid, followed by any other amino acid, are targets for V8 protease secreted by *S. aureus*. Arginine-specific (PR or GR) or lysine-specific (GK or AK) sequences are targets for gingipain (Rgp or Kgp) proteases secreted by *P. gingivalis*, a primary driver of periodontitis. These sequences can be implemented into dental implants or local gels that release the drug only if the bacteria are present in the biofilm.

Enzyme-responsive functional groups represent one of the most selective molecular strategies for targeted drug delivery because enzymatic activity is often restricted to specific tissues, pathological microenvironments, or microorganisms. Unlike physicochemical stimuli such as pH or redox gradients, enzymes recognize well-defined chemical motifs and catalyze highly specific bond cleavage, allowing therapeutic activation to occur only where the corresponding enzyme is expressed.

**Figure 3:**
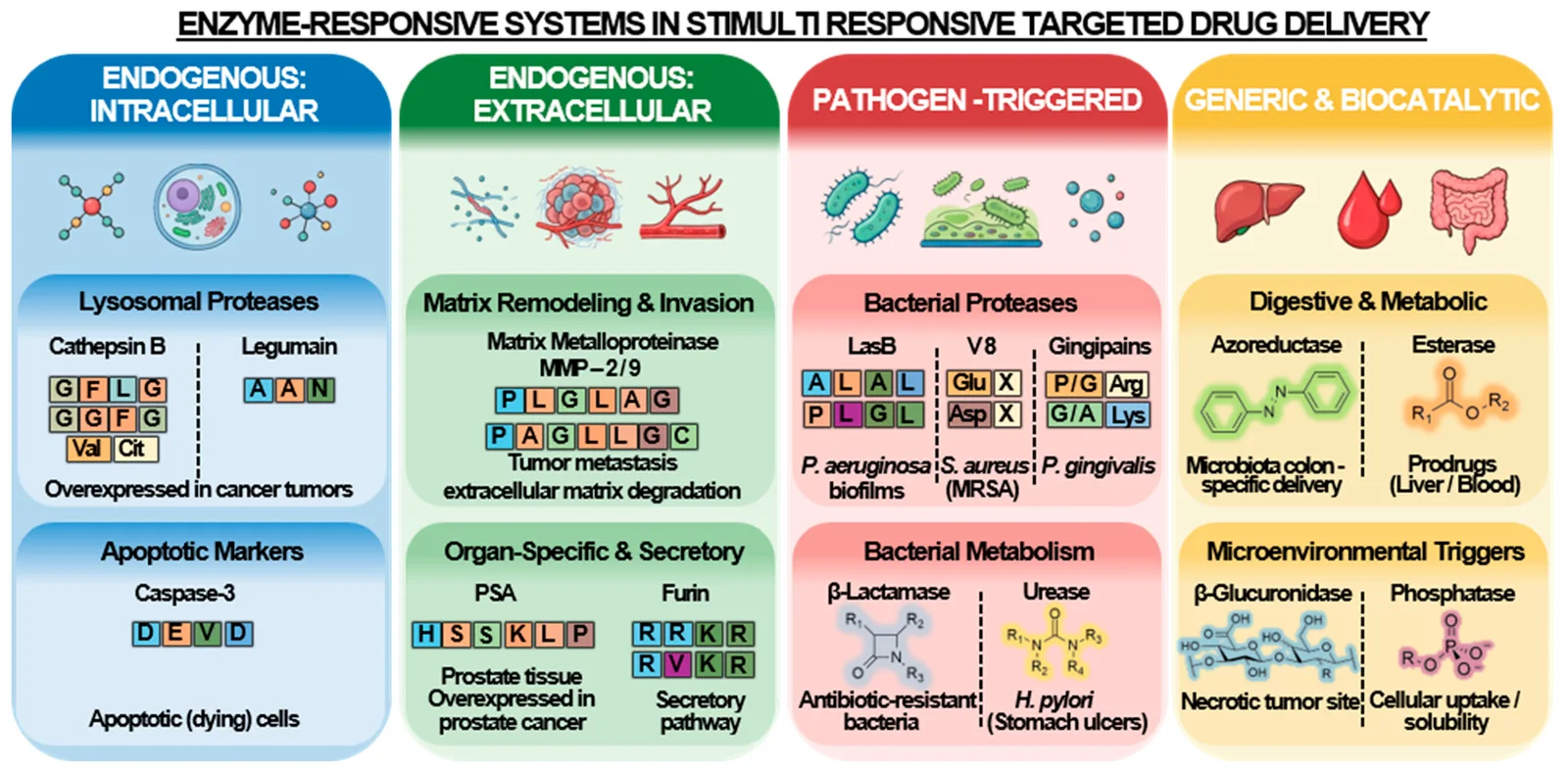
Representative enzyme-responsive functional groups employed in polysaccharide-based drug delivery systems. Intracellular, extracellular, pathogen-associated, and digestive or metabolic enzymes selectively cleave peptide, azo, ester, glycosidic, or phosphate-containing linkages to trigger site-specific drug release. Google Gemini was used to generate the preliminary visual concepts and visual artwork representations as blue-prints for this figure. Prompts outlining chemical linkages, biological pathways, and structural release mechanisms were provided to guide visual creation. The study conception, data interpretation, and final validation of the visual accuracy were exclusively performed by the authors.

Depending on the targeted pathology, these systems exploit intracellular lysosomal enzymes, extracellular proteases involved in tissue remodeling and tumor invasion, pathogen-derived enzymes associated with bacterial infections, or digestive and metabolic enzymes characteristic of particular organs or disease states. The principal enzyme-responsive functional groups and activation pathways discussed in this review are summarized in Figure 3. Enzyme-responsive drug delivery systems exploit the selective overexpression or unique activity of endogenous, pathogen-derived, or metabolic enzymes to achieve highly specific therapeutic activation.

Intracellular enzymes, including lysosomal proteases such as cathepsin B and legumain, as well as apoptotic caspases, cleave peptide-based linkers following cellular internalization, enabling drug release within lysosomes or apoptotic cells. Extracellular enzymes, including matrix metalloproteinases (MMP-2 and MMP-9), prostate-specific antigen (PSA), and furin, recognize disease-associated peptide sequences and trigger drug release within the tumor microenvironment or secretory pathways. Pathogen-associated enzymes, such as bacterial proteases, β-lactamases, and urease, provide selective activation in infectious microenvironments, whereas digestive and metabolic enzymes, including azoreductases, esterases, β-glucuronidase, and phosphatases, cleave azo, ester, glycosidic, or phosphate-containing functional groups to enable colon-specific drug delivery, prodrug activation, or selective release in disease-specific tissues.

Subsequently, these enzyme-responsive mechanisms translate disease-associated enzymatic signatures into highly selective therapeutic activation, providing exceptional spatial specificity while minimizing premature drug release and off-target effects.

### 4.4. Glucose Concentration Gradients

Glucose responsiveness is achieved most prominently through the use of the phenylboronic acid (PBA) functional group [54]. PBA is a Lewis acid that exists in an equilibrium between an uncharged, trigonal planar, hydrophobic state and an anionic, tetrahedral, hydrophilic state. It can react reversibly with *cis*-1,2 or *cis*-1,3 diols, such as those present in glucose, with the boronate ion form binding more favorably due to the geometry of the state. The resulting shift in equilibrium away from the neutrally charged PBA, increases the hydrophilicity of the system and accumulation of negative charges causes osmotic swelling of the nanocarrier and subsequent drug release (e.g., insulin release). A decrease in glucose concentration as a result of the released drug causes the equilibrium to shift back to the neutral state of PBA, causing contraction of the nanocarrier and temporary decrease to the rate of drug release [54].

Boronic acids occupy a unique position among chemically responsive functional groups because they regulate drug release through reversible molecular recognition rather than irreversible bond cleavage. Their ability to selectively bind cis-diol-containing molecules, particularly glucose, enables direct conversion of changes in glucose concentration into predictable physicochemical alterations of the carrier.

**Figure 4:**
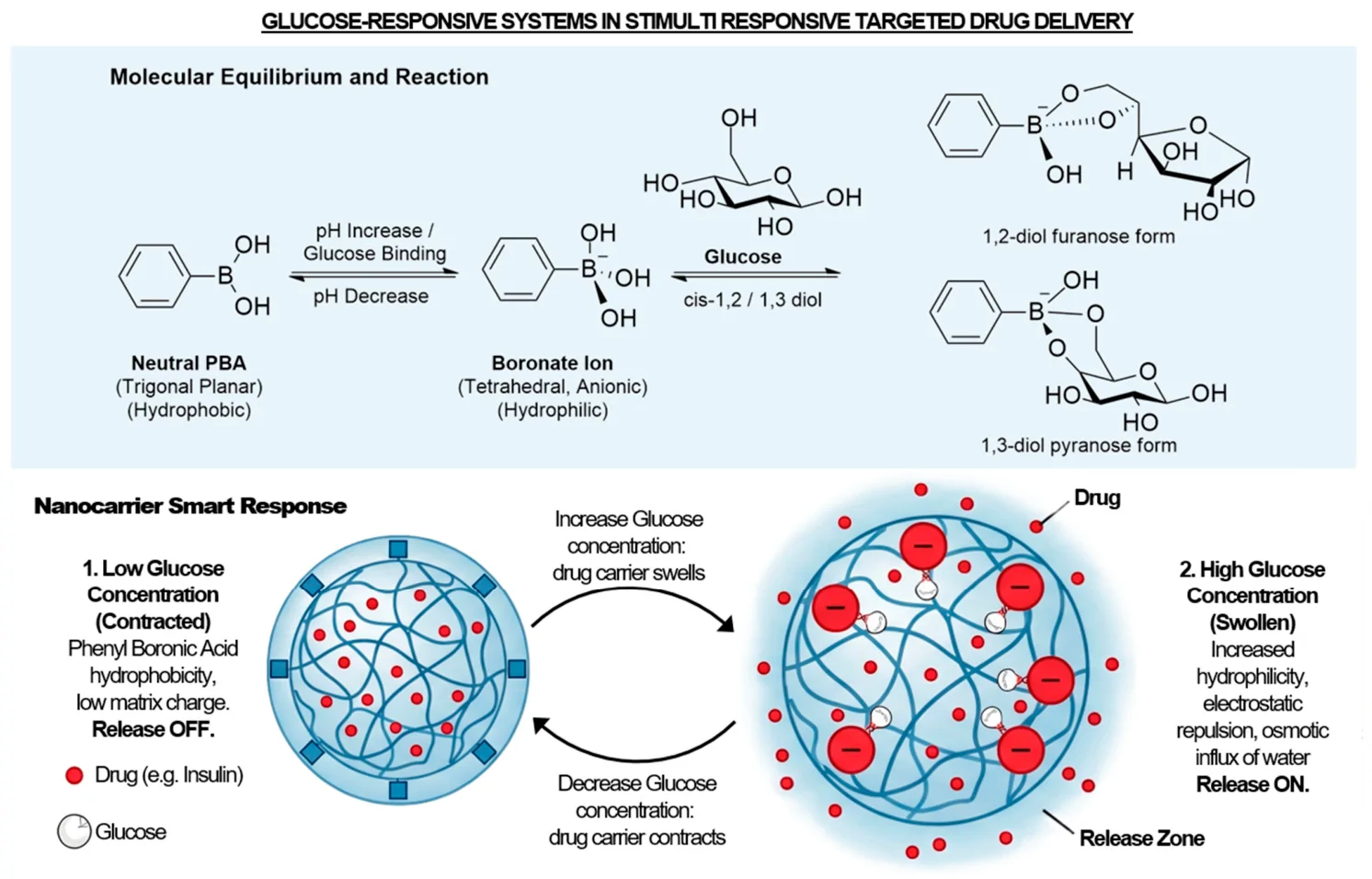
Schematic representation of glucose-responsive drug delivery mediated by phenylboronic acid functional groups. Reversible boronic acid–cis-diol complexation regulates polymer ionization, network swelling, and drug diffusion in response to changes in glucose concentration. Google Gemini was used to generate the preliminary visual concepts and visual artwork representations as blue-prints for this figure. Prompts outlining chemical linkages, biological pathways, and structural release mechanisms were provided to guide visual creation. The study conception, data interpretation, and final validation of the visual accuracy were exclusively performed by the authors.

Formation and dissociation of boronate ester complexes modulate polymer charge, hydrophilicity, and network swelling without degrading the carrier structure, allowing repeated activation cycles. Consequently, boronic acid-functionalized systems have become one of the most extensively investigated platforms for glucose-responsive drug delivery. The principal molecular mechanism underlying boronic acid-mediated glucose responsiveness is summarized in Figure 4.

Phenylboronic acid exists in equilibrium between a neutral trigonal (sp^2^) form and an anionic tetrahedral boronate (sp^3^) species, with the position of this equilibrium being influenced by both pH and glucose concentration. Binding of cis-diol-containing glucose molecules promotes the formation of stable boronate ester complexes, increasing polymer ionization, hydrophilicity, and osmotic swelling. At low glucose concentrations, the polymer network remains relatively contracted, limiting water uptake and drug diffusion. In contrast, elevated glucose levels shift the equilibrium toward boronate ester formation, resulting in increased electrostatic repulsion, network expansion, enhanced permeability, and accelerated release of encapsulated therapeutics such as insulin.

Owing to the reversible nature of boronic acid–diol complexation, these systems can repeatedly respond to fluctuations in glucose concentration, closely mimicking the physiological feedback mechanisms that regulate endogenous insulin secretion. This reversible molecular recognition mechanism distinguishes boronic acids from most other stimuli-responsive functional groups and makes them particularly attractive for the development of self-regulated glucose-responsive drug delivery systems.

### 4.5. Photo-Responsiveness

The incorporation of photoresponsive functional groups into polysaccharide-based biomaterials has emerged as an attractive strategy for achieving externally controlled drug delivery with exceptional spatial and temporal precision. Unlike endogenous stimuli, such as pH variations, enzymatic activity or redox gradients, light provides a non-invasive trigger whose intensity, wavelength, exposure time and irradiation area can be precisely regulated by the user. This unique level of control enables therapeutic release to be activated on demand, minimizing premature drug leakage while allowing localized treatment with reduced systemic side effects.

Depending on the chemical nature of the incorporated moiety, light may induce reversible photoisomerization, irreversible photocleavage or photocrosslinking reactions, each providing a distinct mechanism for regulating therapeutic release [55]. Therefore, the responsiveness of these materials is dictated not by the polysaccharide backbone itself but by the photochemical behaviour of the functional groups engineered into its structure.

Among the various photoresponsive motifs investigated to date, azobenzene derivatives represent the most extensively studied reversible photoswitches [56]. In contrast, *o*-nitrobenzyl and coumarin derivatives mainly operate through photocleavage reactions that induce irreversible structural disruption, whereas cinnamoyl groups undergo UV-induced [2+2] photocycloaddition, leading to controlled network formation through photocrosslinking [57].

### 4.6. Thermal Gradient (Heat Triggered)

While azobenzenes are preponderantly used as photo-responsive functional groups, some azo-initiator derivatives can be exploited as thermo-labile linkers [58]. Additionally, alkoxyamines can also be fine-tuned to cleave homolytically at the C-ON bond at medically relevant temperatures (37–45 °C) [59]. Often, these are incorporated or grafted onto iron oxide nanoparticles that can be heated locally using an alternating magnetic field or NIR-light source. Another thermo-responsive group is the ureidopyrimidone (UPy) molecule. This can form four strong directional hydrogen bonds that are stable at room temperature, but are increasingly dynamic with increasing temperature [60,61]. Integrating these groups into a polymeric backbone allows the formation of reversible H-bonded networks that can undergo gelation-solation cycles, which release the entrapped drug.

While UPy allows formation of nanocarrier gels using reversible intermolecular H-bonding, reversible intramolecularly bound nanocarriers can be obtained using the Diels-Alder reaction between furan and maleimide derivatives [62]. The click reaction forms stable adducts between 20–40 °C while retro-Diels-Alders occurs usually above 60 °C, but it can be lowered by fine-tuning the system.

## 5. Applications of Functional Group Modifications in Polysaccharide Systems for SRTDD

While the aforementioned techniques can be exploited both in natural and synthetic polymeric nanocarrier systems, the following section specifically examines how functional group modifications within polysaccharide architectures enable the design of precisely tuned, stimuli-responsive delivery vehicles.

### 5.1. Schiff-Base

To mitigate the severe systemic toxicity and poor water solubility of the antifungal and antileishmanial drug Amphotericin B (AmB), researchers frequently leverage Schiff-base chemistry to bind the drug directly to oxidized polysaccharide backbones via reversible imine or hydrazone linkages. Representing a foundational example of this approach, Falk et al. conjugated AmB with oxidized arabinogalactan (AG), a highly branched natural polysaccharide with unusually high-water solubility (70% in water) [63]. The processes produced a highly water-soluble formulation with comparable minimum inhibitory concentrations (MICs) to free AmB (0.1 to 0.2 µg/mL) against *Candida albicans* and *Cryptococcus neoformans* but without the hemolytic side-effects and safer than conventional formulations.

Similar results [64] were obtained by Nishi et al. using gum Arabic (90% arabinogalactan) with unreduced (imine) and reduced (amine) forms of AmB conjugates. Both forms retained their anti-fungal (*C. albicans* and *C. neoformans*) and anti-leishmanial properties, displaying lower systemic toxicity and non-hemolytic behavior. Injectable AmB-containing hydrogels were synthesized by Hudson et al. by cross-linking a dextran-aldehyde-AmB conjugate with carboxymethylcellulose-hydrazide through hydrazone bonds [65]. In vitro controlled release studies showed antifungal activity for 11 days, and toxicity towards *C. albicans* for 3 weeks in contact with the gel. The gel showed no apparent tissue toxicity in murine peritoneum while the specimens did not exhibit abnormal behavior during the experiment. The authors argue that such localized delivery could mitigate the toxicity issues from system administration of AmB or similar molecules in a single injection and addresses practical considerations such as treatment options in communities with poor access to sustained intravenous delivery.

Sokolsky-Papkov et al. investigated the toxicity effects of unreacted aldehyde groups in oxidized dextran-AmB imine conjugates [66]. It was found that the toxicity against RAW 264.7 cell lines was reduced at least 15-fold from IC_50_ of 3 µmol/mL aldehydes by reacting the free aldehydes with ethanolamine. The antiparasitic activity against *Leishmania donovani* of dextran-AmB conjugates was not affected by the reaction with ethanolamine. A more general drug release platform using acetylsalicylic acid as model drug was prepared by Zhang et al. In their study [67], they quaternized carboxymethyl chitosan, which formed rapid self-healing hydrogels when injected alongside oxidized hyaluronic acid and 3,3’-dithiobis-(propionohydrazide). The obtained hydrogels had excellent cytocompatibility (98% cell survival) and 96% self-healing rate after 30 min.

Also, the lyophilized hydrogels were loaded with acetyl salicylic acid by the solvent adsorption method. The drug release was studied as a function of pH by lyophilizing the drug-loaded hydrogel and swelling in PBS solutions at different pH values (4, 7.4, 10). The release was fastest (72–85%) in acidic conditions, followed by (52–74%) in neutral conditions and (49–61%) under alkaline conditions. The presence of the quaternary ammonium salts in the chitosan structure provided significant antimicrobial effect, particularly against Gram-positive bacteria (*S. aureus*) compared to Gram-negative bacteria (*E. coli*). Schiff-base chemistry has also been successfully leveraged in anticancer formulations and responsive hydrogels. Patel et al. modified Gemcitabine (GEM) with hydrazine to form an acid labile linker bound to alginate while also synthesizing a chitosan-gemcitabine conjugated linked through a dithio-di-glycolic acid molecule [68]. The two polysaccharide-gemcitabine conjugates were used to form nanoparticles through polyelectrolyte complexation of the negatively charged alginate and positively charged chitosan. The nanoparticles exhibited dual stimuli-responsiveness from the pH labile hydrazone group and redox responsiveness to glutathione (GSH) from the dithio- group. The nanoplatform’s therapeutic potential was evaluated against 4T1, MCF-7 and MDA-MB-231 breast cancer cell lines showing increased cellular uptake, enhanced cytotoxicity and efficient apoptosis. In vivo studies on mice bearing 4T1-Luc tumor validated the in vitro results, exhibiting tumor growth inhibition at 10 mg/kg GEM equivalent dose, with reduced toxicity compared to free GEM.

Another formulation with potential anticancer properties was developed by Yang et al. [69], in which self-healing hydrogels based on modified cellulose and poly(ethylene glycol) (PEG) were used to prove controlled released of doxorubicin. Cellulose was conjugated, at the carboxyl group, with dithiodipropionate dihydrazide, while PEG was capped with 4-hydroxybenzaldehyde, allowing crosslinking through hydrazone bonds formation. The hydrogels displayed dual pH/redox responsive sol-gel transitions, high-healing efficiencies in a 4-amino-DL-phenylalanine concentration dependency (96%) and high biocompatibility. It was also shown that the hydrogel can serve as a 3D cell culture platform as evidenced by high viability and proliferation of L929 cells.

Similar studies were carried out by Alkanawati et al. on model release cargoes from nanogels with multiresponsive release, using the pH-responsiveness of hydrazone linkages, the enzymatic cleavage of the dextran backbone and incorporation of disulfide groups and thioketal groups for redox responsiveness [70,71]. Dextran was modified in a two-step process to introduce aldehyde groups by oxidation and introduction of ketone groups by reaction with levulinic acid. Dextran nanogels were crosslinked with adipic acid dihydrazide, thioketal dihydrazide or 3,3′-dithiodipropionic acid dihydrazide or blends of crosslinkers. The authors evaluated the loading and release of FITC-albumin as a model macromolecule, observing no dependency on the cross-linker used on the loading efficiency. The release studies showed a reversible swelling process going from neutral pH to acidic buffer, described as an on–off release of cargo when the buffer was returned to pH 7.4.

Hyaluronic acid modified with adipic acid dihydrazide by Cai et al. and Yin et al. has been successfully conjugated with doxorubicin for improved survivability and reduced toxicity in cancer treatment [72,73]. The release half lives of doxorubicin via Schiff base mechanism were shown to be pH-dependent, with a ratio of 1:2.4:3.8 at pH 5.0, 6.0 and 7.4. In rodent models, the conjugate system greatly reduced cardiac toxicity, with only 20% animals developing myocyte degeneration, compared to 83% that developed myocarditis in the free doxorubicin single-dose injection. Yin et al. [73] further showed that introduction of redox-responsiveness via cystamine-modified hyaluronic acid for a dual-responsive system (low pH and high GSH concentration) improved the therapeutic effect of doxorubicin at the tumor site.

pH-sensitive hydrogels based on hydrazones were also prepared by crosslinking oxidized dextran with polyhydrazides for the delivery of hydrophilic anticancer drug 5-fluorouracil (5-FLU) as shown by Jalalvandi et al. [74]. The gels showed no toxicity towards L929 mouse fibroblast cells, and the release of 5-FLU was through diffusion mechanisms. The gel degradation and release kinetics was shown to be dependent on the cross-linking density and the pH of the environment, with lower pH favoring hydrazone bond hydrolysis.

Hydrazone-functionalized polysaccharides can also be used for applications such as biodegradable barriers for use post-surgery. Yeo et al. cross-linked adipic dihydrazide-modified hyaluronic acid with aldehyde modified hyaluronic acid to form in situ hyaluronic acid hydrogels that formed a biodegradable physical barrier against post-operative peritoneal adhesions in rabbit model [75]. In vitro cytotoxicity studies of hyaluronic acid gels on mesothelial cells showed that the gels had no significant effect on cell viability except for a minor decrease in the presence of hyaluronidase, which degrades the gels. In vivo studies showed that only 25% of samples treated with the hyaluronic acid gel developed adhesions compared to 83% of samples not treated.

### 5.2. Acetal- and Ketal-Functionalized Polysaccharides: Acid-Triggered Bond Cleavage for Controlled Drug Delivery

Acetal- and ketal-containing functional groups represent one of the most established molecular strategies for engineering acid-responsive polysaccharide-based drug delivery systems. Their widespread use arises from a unique combination of chemical stability under physiological conditions and rapid hydrolysis in mildly acidic environments, enabling selective release of therapeutics within tumor tissues, inflamed sites, endosomes, and lysosomes. Unlike ionizable polymers, whose response relies on reversible swelling or contraction, acetal and ketal systems regulate drug release through the cleavage of covalent bonds. Protonation of the acetal oxygen under acidic conditions initiates hydrolysis, regenerating the parent hydroxyl groups while simultaneously disrupting the polymer network or releasing the conjugated therapeutic.

This molecular mechanism enables precise temporal control over cargo release while maintaining excellent stability during circulation.

Among naturally derived polysaccharides, acetalated dextran (Ac-DEX) has become the benchmark material for acid-responsive delivery systems. Ac-DEX is synthesized by reacting dextran hydroxyl groups with 2-methoxypropene under mild acid catalysis, producing a hydrophobic polymer containing both cyclic and acyclic acetal groups. The acetal modification transforms water-soluble dextran into an organic-soluble material that can be readily processed into microparticles and nanoparticles using conventional emulsion techniques.

Following exposure to acidic environments, hydrolysis of the pendant acetal groups restores the native hydrophilic dextran backbone while generating acetone and methanol (or ethanol in Ac-DEX analogues) as low-toxicity degradation products, providing a biocompatible degradation pathway [76,77].

A defining characteristic of Ac-DEX is the possibility of programming degradation kinetics through the relative proportion of cyclic and acyclic acetal groups. During synthesis, short reaction times favor the formation of kinetically controlled acyclic acetals, whereas prolonged reactions increase the fraction of thermodynamically more stable cyclic acetals. Consequently, degradation rates can be tuned over several orders of magnitude without altering the polymer composition itself.

Broaders et al. [77] first demonstrated that this tunability directly influences antigen presentation pathways following encapsulation of protein antigens, while later studies showed that degradation profiles may be further adjusted by controlling both cyclic acetal coverage and dextran molecular weight. Polymers containing lower cyclic acetal content or higher molecular-weight dextran exhibited faster hydrolysis, accelerated cargo release, and enhanced biological activity compared with more slowly degrading formulations [78].

The molecular control of degradation offered by Ac-DEX has enabled its application across a broad spectrum of therapeutic platforms. In cancer nanomedicine, Ac-DEX nanoparticles have been successfully employed for the encapsulation of platinum(IV) prodrugs together with near-infrared fluorophores, generating theranostic nanoparticles that combine imaging with controlled chemotherapy. These nanoparticles remained highly stable at physiological pH (7.4) while undergoing rapid degradation at pH 5.5, resulting in complete and sustained release of the encapsulated platinum prodrug following endosomal uptake. Importantly, encapsulation significantly enhanced cytotoxicity compared with the free Pt(IV) precursor, illustrating how acid-triggered degradation improves intracellular drug bioavailability [79].

Recent developments have further extended the concept of programmable release beyond simple ON-state activation. Erensoy et al. [80] demonstrated that nanoparticles prepared by mixing Ac-DEX polymers possessing different degradation rates produced highly responsive formulations capable of reversible ON/OFF drug release. These hybrid nanoparticles rapidly initiated cargo release upon transition from physiological pH to the mildly acidic inflammatory microenvironment (pH ~6.0) while autonomously suppressing release when physiological pH was restored. Such dynamic regulation illustrates that carefully tailoring acetal composition allows the release process to follow transient pathological changes rather than simply responding to a single degradation event, representing an important step toward precision inflammatory therapy.

Although Ac-DEX represents the most extensively investigated acetal-functionalized polysaccharide, similar molecular principles have also been applied to other natural polymers. Hyaluronic acid nanogels crosslinked with ketal-containing crosslinkers have demonstrated accelerated doxorubicin release under acidic conditions while maintaining excellent stability at physiological pH. Besides the acid-triggered degradation of ketal crosslinks, these systems benefit from the intrinsic affinity of hyaluronic acid for CD44-overexpressing tumor cells, combining active targeting with pH-responsive intracellular release. Enhanced cellular uptake, increased nuclear accumulation of doxorubicin, and superior antitumor efficacy compared with non-responsive nanogels confirmed the advantages of integrating ketal chemistry into naturally occurring polysaccharides [81].

Overall, acetal- and ketal-functionalized polysaccharides exemplify how modification of a single functional group can precisely dictate the molecular mechanism of therapeutic release. Rather than relying on polymer swelling or enzymatic degradation, these systems employ predictable acid-catalyzed cleavage of covalent bonds to convert stable carriers into rapidly degradable matrices within pathological microenvironments. Furthermore, the possibility of independently tuning degradation kinetics through acetal composition, molecular weight, or crosslink density provides an exceptional level of control over release profiles. These characteristics establish acetal and ketal chemistry as one of the most mature and versatile molecular engineering strategies for stimuli-responsive polysaccharide drug delivery and provide a valuable conceptual framework for the development of future self-regulated and self-reporting therapeutic delivery systems.

### 5.3. Boronate Ester Linkages: Cis-Diol Chemistry for Glucose and ROS Responsiveness

Boronate ester chemistry has emerged as one of the most versatile dynamic covalent strategies for engineering stimuli-responsive polysaccharide-based drug delivery systems.

Unlike conventional covalent crosslinks, boronate esters are formed through the reversible complexation of boronic acid moieties with cis-diol-containing molecules, generating dynamic covalent bonds capable of continuously breaking and reforming under physiological conditions. This unique molecular recognition process enables polymer networks to reversibly adapt their architecture in response to multiple biochemical stimuli, including glucose concentration, ROS, and pH variations.

Consequently, boronic acid-functionalized polysaccharides have attracted considerable attention as intelligent biomaterials capable of coupling disease-associated molecular signals with precisely controlled therapeutic release while simultaneously providing injectability, self-healing, shear-thinning behavior, and mechanical adaptability.

Comprehensive overviews of boronic acid chemistry have highlighted that the unique combination of reversible covalent exchange and selective molecular recognition distinguishes boronate esters from most other dynamic functional groups currently employed in responsive drug delivery systems [82,83].

The dynamic behavior of boronate esters originates from the Lewis acidity of boronic acids. In aqueous media, boronic acids exist in equilibrium between a neutral trigonal planar form and a tetrahedral boronate species. Above their apparent pKa, boronic acids readily react with 1,2- or 1,3-*cis*-diols to form cyclic boronate esters, whereas decreasing pH shifts the equilibrium toward hydrolysis and dissociation of these dynamic covalent bonds. The exact position of this equilibrium depends on the electronic characteristics of the boronic acid substituents, allowing fine modulation of binding affinity under physiological conditions.

Besides reversible hydrolysis, arylboronic acids undergo selective oxidation by hydrogen peroxide and peroxynitrite to generate phenolic derivatives, producing irreversible cleavage of the boronate linkage. Furthermore, glucose and other saccharides compete directly with polymer-bound cis-diols for boronic acid binding, thereby disrupting existing crosslinks through competitive complexation. Since hyperglycemia and oxidative stress represent characteristic hallmarks of diabetes, chronic inflammation, bacterial infection, and cancer, boronate ester chemistry offers a direct molecular mechanism through which pathological biochemical signals can regulate carrier degradation, network reorganization, and therapeutic release [82].

Among naturally occurring polysaccharides, hyaluronic acid has become one of the most extensively investigated platforms for exploiting dynamic boronate ester chemistry. Initial studies employed phenylboronic acid-functionalized hyaluronic acid together with complementary saccharide-modified hyaluronic acid derivatives to generate injectable hydrogels through spontaneous boronate ester formation under physiological conditions.

Owing to the continuous association and dissociation of these dynamic covalent crosslinks, the resulting hydrogels displayed rapid self-healing, shear-thinning behavior, injectability, and efficient recovery of their mechanical properties following repeated deformation. Such characteristics overcome one of the principal limitations of permanently crosslinked hydrogels, namely their inability to reorganize after mechanical damage, while maintaining sufficient structural integrity for biomedical applications.

Figueiredo et al. [84] further improved this strategy by replacing conventional phenylboronic acid with benzoxaborin derivatives, whose lower pKa and stronger affinity toward carbohydrates enable efficient crosslinking at physiological pH. The resulting hydrogels exhibited enhanced mechanical stability, rapid structural recovery after injection, and sustained viability of encapsulated fibroblasts, illustrating how subtle modifications of boronic acid chemistry can markedly improve the performance of injectable polysaccharide biomaterials.

The versatility of boronate ester chemistry has subsequently been extended beyond hyaluronic acid to alginate-based systems, demonstrating that dynamic cis-diol recognition can simultaneously regulate structural organization, mechanical properties, and biological performance. Hong et al. [85] developed alginate functionalized with phenylboronic acid as a single-component hydrogel exhibiting an exceptional combination of stretchability, self-healing, shear-thinning behavior, glucose responsiveness, pH sensitivity, remoldability, and adhesive properties. Unlike conventional multifunctional hydrogels that require several independent crosslinking mechanisms, the remarkable adaptability of this material originated exclusively from the reversible equilibrium between boronic acid and the cis-diol groups naturally present along the alginate backbone. Single-molecule force spectroscopy further confirmed that the continuous formation and dissociation of boronate ester bonds governs the reconstruction of the polymeric network, thereby providing a molecular explanation for the observed macroscopic self-healing and mechanical resilience.

Building upon the same molecular concept, Hong et al. [86] subsequently introduced an alginate–boronic acid bioinspired adhesive capable of assembling independent hydrogel building blocks through reversible cis-diol interactions. Rather than functioning as a conventional crosslinked scaffold, this system behaves as a dynamic molecular glue in which polymer diffusion across hydrated interfaces precedes boronate ester formation following a slight increase in pH. The resulting adhesive enables robust integration of chemically distinct hydrogels while preserving their individual biological characteristics, thereby expanding the application of boronate ester chemistry from responsive drug delivery toward modular tissue engineering and hydrogel assembly. These studies collectively demonstrate that the dynamic nature of boronate ester exchange can be exploited not only to regulate therapeutic release but also to engineer mechanically adaptive polysaccharide biomaterials capable of autonomous structural reorganization under physiological conditions.

Beyond hydrogel formation, boronate ester chemistry has been widely exploited for constructing nanoscale polysaccharide carriers in which dynamic covalent crosslinks regulate cargo loading and controlled release. One of the earliest demonstrations was reported by Li et al., who functionalized dextran with phenylboronic acid to obtain amphiphilic polymers that spontaneously self-assembled into nanoparticles capable of encapsulating doxorubicin [87]. Under physiological conditions, hydrophobic boronate ester linkages stabilized the nanoparticle structure, whereas exposure to mildly acidic environments promoted hydrolysis of the boronate esters, restoring the hydrophilic character of dextran and triggering particle disassembly accompanied by sustained drug release. This study established that reversible boronate ester formation could simultaneously control nanoparticle assembly, structural stability, and pH-responsive therapeutic delivery using a naturally derived polysaccharide backbone.

More recently, Abdi et al. expanded this concept by developing entirely polysaccharide-based nanogels assembled through reversible boronate ester formation between phenylboronic acid-modified dextran and glycopolymer derivatives [88]. Unlike permanently crosslinked nanocarriers, these systems retained their structural integrity under physiological conditions while remaining highly responsive to competitive glucose binding and oxidative environments. The dynamic nature of the boronate ester junctions enabled reversible swelling, controlled degradation, and efficient release of encapsulated therapeutics without requiring additional synthetic crosslinkers or chemically inert components. This fully polysaccharide architecture highlights how boronate ester chemistry can generate nanocarriers that combine excellent biocompatibility with programmable responsiveness toward multiple biologically relevant stimuli.

Among the various external triggers capable of regulating boronate ester exchange, glucose has received particular attention because of its direct clinical relevance to diabetes mellitus. Elevated glucose concentrations compete with polymer-bound cis-diols for boronic acid binding, progressively disrupting boronate ester crosslinks and increasing network permeability. This competitive molecular recognition process allows drug release to become directly proportional to glucose concentration, providing an elegant mechanism for autonomous therapeutic regulation. Exploiting this principle, Meng et al. developed shape-memory polysaccharide hydrogels in which reversible boronate ester crosslinks served both as dynamic structural junctions and glucose-sensitive molecular switches [89], Exposure to physiological glucose concentrations gradually destabilized the polymer network, enabling controlled structural recovery and stimulus-dependent release while preserving the hydrogel’s excellent self-healing capability.

The same group subsequently demonstrated that the reversible nature of boronate ester exchange could be integrated with hierarchical hydrogel architectures exhibiting macroscopic and microscopic shape-memory behavior [90]. Rather than functioning solely as responsive crosslinkers, boronate ester bonds continuously reorganized throughout the polymer network, allowing repeated deformation, autonomous structural recovery, and reversible switching between temporary and permanent configurations under sugar- or pH-controlled conditions. These studies illustrate that glucose-responsive drug delivery is not simply a consequence of network degradation but results from continuous dynamic remodeling of the polymer architecture, which simultaneously governs mechanical adaptation, molecular diffusion, and release kinetics.

In addition to glucose responsiveness, oxidative stress has emerged as an equally important trigger for boronate ester-mediated drug delivery. Hydrogen peroxide, which accumulates in inflamed tissues, infected wounds, and solid tumors, selectively oxidizes arylboronic acids into phenolic derivatives, irreversibly cleaving boronate ester crosslinks and accelerating degradation of the polymer network. Because hydrogen peroxide concentrations are typically several orders of magnitude higher in pathological environments than in healthy tissues, ROS-responsive boronate systems provide substantially greater biological specificity than conventional pH-responsive carriers. Hu et al. exploited this mechanism by engineering injectable alginate-based hydrogels incorporating phenylboronic acid functionalities that responded simultaneously to acidic pH and elevated ROS concentrations [91]. The dual-responsive matrix enabled localized release of encapsulated therapeutic agents under inflammatory conditions while maintaining high structural stability under physiological environments, thereby accelerating tissue repair and reducing premature drug leakage.

The potential of combining multiple dynamic chemistries within a single polysaccharide platform was further demonstrated by Balakrishnan et al., who integrated reversible boronate ester formation with dynamic imine bonds to construct tumor-responsive injectable prodrug hydrogels [92]. In this system, each dynamic covalent interaction responded to distinct pathological stimuli, enabling synergistic regulation of network stability, mechanical adaptability, and localized doxorubicin release. The coexistence of complementary reversible chemistries significantly enhanced control over degradation kinetics while preserving injectability, self-healing behavior, and prolonged retention at the administration site. These multifunctional designs exemplify the growing trend toward integrating boronate ester chemistry with other dynamic covalent reactions to generate increasingly sophisticated polysaccharide drug delivery platforms capable of responding simultaneously to multiple biochemical signals characteristic of diseased tissues.

Beyond their role as responsive crosslinkers, boronate esters also provide an effective strategy for simultaneously tailoring the structural, mechanical, and biological properties of polysaccharide biomaterials. This multifunctionality arises from the continuous exchange of dynamic B–O bonds, which enables polymer networks to dissipate mechanical stress through reversible bond rearrangement while maintaining overall structural integrity. Consequently, boronate ester-crosslinked hydrogels frequently combine injectability, self-healing, shear-thinning behavior, moldability, and stimuli-responsive drug release within a single material platform, eliminating the need for multiple independent crosslinking chemistries.

The versatility of this approach has been demonstrated using cellulose, one of the most abundant natural polysaccharides employed in biomedical engineering. An et al. developed cellulose-based hydrogels functionalized through dynamic boronate ester chemistry that exhibited rapid autonomous self-healing, excellent mechanical robustness, and sustained release of doxorubicin while maintaining high cytocompatibility [93]. Owing to the reversible nature of the boronate ester junctions, damaged hydrogels rapidly recovered their structural integrity without external intervention, illustrating how dynamic covalent chemistry can simultaneously improve mechanical reliability and therapeutic performance. Importantly, these materials retained their responsiveness under physiological conditions, further supporting the suitability of boronate ester-functionalized cellulose as a platform for injectable and implantable drug delivery systems.

A complementary strategy was reported by Ge et al., who combined cellulose nanofibers with poly(vinyl alcohol), borax, and tannic acid to fabricate multifunctional hydrogels possessing excellent mechanical strength, antioxidant activity, self-healing capability, and tissue adhesiveness [94]. Although the material incorporated additional supramolecular interactions, reversible boronate ester formation remained the principal mechanism governing dynamic network reconstruction and mechanical recovery. This work highlights an important evolution in boronate ester chemistry, where dynamic B–O bonds are no longer used solely as stimuli-responsive crosslinks but are integrated with complementary intermolecular interactions to produce multifunctional biomaterials capable of addressing several clinical requirements simultaneously, including structural stability, localized drug delivery, and wound healing.

Collectively, these studies demonstrate that boronate ester chemistry has evolved from a simple glucose-responsive molecular recognition strategy into a comprehensive platform for engineering adaptive polysaccharide biomaterials. Depending on polymer composition and boronic acid structure, reversible B–O bond formation can regulate hydrogel assembly, nanoparticle self-organization, mechanical resilience, injectability, self-healing, and disease-triggered therapeutic release through a common dynamic covalent mechanism. Furthermore, the ability of boronate esters to respond selectively to biologically relevant stimuli—including glucose, pH, and reactive oxygen species—provides a direct molecular interface between pathological biochemical signals and controlled drug delivery. Rather than functioning merely as reversible crosslinkers, boronate ester linkages therefore serve as dynamic molecular regulators that integrate structural adaptation with therapeutic function. Their exceptional chemical versatility, combined with the intrinsic biocompatibility of polysaccharides, positions boronate ester chemistry among the most powerful functional-group strategies currently available for the rational design of next-generation smart drug delivery systems.

### 5.4. Cis-Aconityl Linkages: Acid-Labile Spacers for Intracellular Drug Release

The cis-aconityl linker is one of the earliest and most extensively investigated acid-labile spacers for engineering pH-responsive polymer–drug conjugates. Unlike ionizable polysaccharides, which regulate therapeutic release through reversible swelling and contraction, or acetal/ketal linkages, which rely on hydrolysis of dynamic covalent bonds within the carrier, cis-aconityl functions primarily as a cleavable molecular spacer between the polysaccharide backbone and the therapeutic agent. The linker remains highly stable under physiological conditions (pH 7.2–7.4) but undergoes rapid acid-catalyzed hydrolysis within the mildly acidic environments of tumors, endosomes, and lysosomes (pH 5.0–6.5), enabling intracellular drug liberation while minimizing premature release during systemic circulation [95,96].

The molecular mechanism responsible for drug release originates from the protonation of the cis-aconityl amide bond under acidic conditions. Proton-assisted cleavage destabilizes the amide linkage connecting the drug molecule to the polymer, regenerating free doxorubicin while leaving the polysaccharide carrier largely intact. Consequently, the release process is dictated by the hydrolysis kinetics of the spacer rather than degradation of the polymer matrix itself. Molecular modeling studies further demonstrated that cis-aconityl conjugation significantly stabilizes the drug–polymer complex under neutral conditions while substantially lowering the activation barrier for drug release in acidic media. Computational simulations performed on glycol chitosan–doxorubicin conjugates indicated that drug liberation becomes energetically favorable only under acidic conditions, whereas the corresponding reaction is highly unfavorable at physiological pH. Moreover, the length of polyethylene glycol spacers influenced both conjugate stability and release kinetics, suggesting that molecular architecture can further regulate intracellular drug liberation [97].

Among naturally occurring polysaccharides, chitosan has become one of the preferred platforms for cis-aconityl-mediated drug conjugation owing to its abundant amino groups, excellent biocompatibility, and favorable intracellular trafficking. Hu et al. developed stearic acid-grafted chitosan oligosaccharide micelles covalently linked to doxorubicin through cis-aconityl bonds [95]. The amphiphilic conjugates self-assembled into nanosized micelles exhibiting excellent colloidal stability, low critical micelle concentration, and pronounced pH-responsive release. While drug leakage remained minimal at physiological pH, decreasing the pH from 7.2 to 5.0 markedly accelerated doxorubicin release. Importantly, the conjugated micelles effectively overcame multidrug resistance in MCF-7/Adr breast cancer cells and demonstrated significantly enhanced antitumor efficacy together with reduced systemic toxicity in vivo compared with free doxorubicin, illustrating how acid-labile molecular linkers can simultaneously improve therapeutic selectivity and pharmacological performance.

A complementary strategy employed glycol chitosan as the carrier backbone. By combining the hydrophilicity of glycol chitosan with cis-aconityl-mediated conjugation, stable polymer–drug conjugates were obtained that minimized premature drug leakage during circulation while preserving rapid intracellular release after endocytosis. Computational investigations indicated that increasing PEG chain length enhanced conjugate stability, whereas diethylene glycol spacers provided the most favorable balance between structural integrity and efficient drug release. These observations highlight that optimization of both the acid-labile linker and the surrounding polymer architecture contributes to precise control of intracellular drug delivery [97].

The cis-aconityl strategy has also been successfully integrated into hyaluronic acid, combining acid-responsive release with receptor-mediated tumor targeting. Lin et al. developed amphiphilic hyaluronic acid nanoparticles in which doxorubicin was conjugated through cis-aconityl linkages, while disulfide bonds introduced an additional redox-responsive mechanism [96]. Following self-assembly, the nanoparticles remained stable during circulation but underwent accelerated drug release under the combined influence of acidic pH and elevated intracellular glutathione concentrations. Incorporation of luteinizing hormone-releasing hormone (LHRH) peptides further enhanced selective uptake by ovarian cancer cells, whereas the intrinsic affinity of hyaluronic acid for CD44 receptors promoted active tumor targeting. Nearly complete drug release was achieved within approximately 150 h under intracellular conditions, resulting in substantial inhibition of orthotopic ovarian tumor growth together with simultaneous near-infrared imaging capability [96].

More recently, Zhang et al. extended this concept by integrating cis-aconityl-mediated drug release into a multifunctional hyaluronic acid nanoplatform carrying both a nitric oxide (NO) prodrug and a cis-aconityl-linked doxorubicin prodrug [98]. The resulting nanoparticles implemented a relay delivery strategy consisting of NO-mediated extracellular matrix remodeling, CD44-mediated tumor targeting, and tumor microenvironment-responsive release. While glutathione and glutathione-S-transferase triggered sustained NO liberation, the combination of acidic pH and intracellular glutathione accelerated cleavage of the cis-aconityl linker, releasing doxorubicin specifically within tumor cells. The synergistic action of NO and chemotherapy increased oxidative stress, enhanced nanoparticle penetration into dense tumor tissue, and produced tumor inhibition rates approaching 88% in vivo, demonstrating how cis-aconityl chemistry can be integrated into sophisticated multistage delivery systems [98].

Beyond soft polymeric nanoparticles, cis-aconityl linkers have also been incorporated into rigid polysaccharide nanomaterials. Li et al. functionalized rod-like cellulose nanocrystals with cis-aconityl-doxorubicin conjugates, producing fluorescent nanorods capable of simultaneously serving as imaging probes and intracellular drug carriers [99]. The cellulose nanocrystals preserved their rod-like morphology after functionalization and exhibited enhanced cellular uptake compared with free doxorubicin. Drug release remained limited at physiological pH but increased progressively as pH decreased from 7.4 to 5.0, reaching approximately 80% cumulative release after 40 h. Lysosomal alkalization using ammonium chloride markedly reduced drug liberation, confirming that cleavage of the cis-aconityl spacer was driven primarily by the acidic lysosomal environment. Furthermore, the intrinsic fluorescence of doxorubicin enabled direct visualization of intracellular trafficking without additional fluorescent labels, illustrating the potential of cis-aconityl systems for image-guided drug delivery.

Collectively, these studies establish cis-aconityl as one of the most reliable acid-labile molecular linkers for polysaccharide-based drug delivery. Unlike functional groups that regulate release through reversible swelling or network disassembly, cis-aconityl provides direct molecular control by selectively cleaving the polymer–drug connection under intracellular acidic conditions. Its successful incorporation into chitosan, glycol chitosan, hyaluronic acid, and cellulose nanocrystals demonstrates broad compatibility with natural polysaccharides while enabling precise spatial control of therapeutic release. Within the context of engineering release at the molecular level, cis-aconityl chemistry exemplifies how careful molecular design of a single cleavable spacer can transform passive polymer carriers into highly selective intracellular delivery systems. Moreover, the recent integration of cis-aconityl linkers into multifunctional and theranostic nanoplatforms suggests that this strategy will remain an important foundation for the development of next-generation stimuli-responsive polysaccharide drug delivery systems.

### 5.5. Ionizable Backbone Groups: pH-Driven Swelling and Contraction in Polysaccharide-Based Drug Delivery Systems

The incorporation of ionizable functional groups into polysaccharide backbones represents one of the most extensively investigated strategies for engineering pH-responsive drug delivery systems. Unlike acid-labile covalent linkages, where drug release is initiated by bond cleavage, ionizable polysaccharides regulate therapeutic release through reversible protonation and deprotonation processes that alter electrostatic interactions, osmotic pressure, and polymer chain conformation. Consequently, changes in environmental pH induce reversible swelling or contraction of the polymer network, modulating mesh size, diffusion pathways, and ultimately the release kinetics of encapsulated therapeutics [100,101,102].

The molecular mechanism underlying these systems is governed by the ionization state of acidic or basic functional groups naturally present or chemically introduced into polysaccharides. Carboxyl-containing polysaccharides, including alginate, hyaluronic acid, pectin, carboxymethyl cellulose, and oxidized dextran derivatives, become increasingly ionized as pH rises above their pKa values. Electrostatic repulsion between negatively charged carboxylate groups expands the polymer chains, resulting in enhanced swelling, larger pore dimensions, and accelerated diffusion of incorporated drugs. Conversely, under acidic conditions protonation suppresses electrostatic repulsion, producing a denser network with reduced permeability. Chitosan exhibits the opposite behavior because of its primary amino groups, which become protonated in acidic environments, increasing chain hydration and swelling, whereas deprotonation at neutral or alkaline pH decreases electrostatic repulsion and modifies drug diffusion pathways [100,103,104].

Among polysaccharides, dextran has become one of the most versatile platforms for pH-responsive drug delivery owing to its excellent biocompatibility and the ease with which hydroxyl groups can be chemically modified. Numerous dextran-based hydrogels, nanoparticles, and nanogels have been engineered by introducing ionizable moieties or grafting synthetic pH-responsive polymers onto the dextran backbone, allowing precise control over swelling behavior and sustained drug release [103]. A representative example is the dextran–glucoxylan composite hydrogel crosslinked with citric acid, which exhibited negligible swelling under simulated gastric conditions (pH 1.2) but extensive swelling at intestinal pH (6.8–7.4). This reversible swelling–deswelling behavior translated into pH-dependent diclofenac release following non-Fickian diffusion kinetics, demonstrating the suitability of ionizable polysaccharide matrices for oral site-specific drug delivery [105]. Similarly, dextrin-based hybrid hydrogels containing methacrylic acid displayed a nearly eightfold increase in equilibrium swelling when the pH increased from 1.2 to 7.4, enabling sustained release of antimicrobial agents while maintaining excellent cytocompatibility [106].

Chitosan remains the most extensively studied cationic polysaccharide for pH-responsive delivery because its protonatable amino groups provide intrinsic responsiveness without requiring extensive chemical modification. Recent studies have expanded this concept by combining chitosan with complementary ionizable polymers to produce semi-interpenetrating polymer networks capable of finely regulating drug release. For example, semi-IPN nanoparticles based on chitosan–poly(methacrylic acid) and chitosan–poly(vinyl imidazole) exhibited excellent biocompatibility together with sustained pH-dependent doxorubicin release governed by reversible protonation and deprotonation of the polymer chains [107]. Likewise, biodegradable succinoglycan/chitosan polyelectrolyte hydrogels displayed marked pH-controlled release of 5-fluorouracil, increasing drug liberation from approximately 60% at physiological pH to nearly complete release under acidic conditions while simultaneously providing antibacterial activity and favorable mechanical properties [108]. These examples illustrate how combining oppositely charged polysaccharides generates dynamic electrostatic networks whose swelling behavior can be tuned by the surrounding ionic environment rather than by irreversible degradation of the polymer matrix.

An alternative strategy relies on dynamic covalent networks that combine ionizable backbones with reversible crosslinking chemistry. Oxidized hydroxypropyl cellulose/carboxymethyl chitosan hydrogels containing dynamic imine linkages exhibited injectable and self-healing behavior while maintaining pronounced pH-responsive release, with significantly faster liberation of phenylalanine at the mildly acidic extracellular pH characteristic of tumor tissues than under physiological conditions [109].

Beyond dextran and chitosan, numerous naturally occurring polysaccharides have been engineered into pH-responsive matrices by exploiting their intrinsic ionizable functional groups or through simple chemical modifications that enhance electrostatic responsiveness. Alginate, hyaluronic acid, pectin, agarose, guar gum, pullulan, and maltodextrin have all demonstrated the ability to regulate drug release through reversible swelling and contraction rather than irreversible polymer degradation. In these systems, the ionization state of carboxyl or amino groups determines polymer hydration, pore size, and diffusion rates, allowing the release profile to adapt continuously to changes in the surrounding microenvironment [100,102,110].

Alginate-based systems illustrate how backbone ionization can be combined with complementary polysaccharides to generate highly tunable delivery platforms. Chondroitin sulfate/alginate-graft-poly(acrylic acid) hydrogels exhibited significantly greater swelling and ketorolac release at pH 7.4 than under acidic conditions, reflecting the increased ionization of carboxylate groups and the resulting expansion of the polymer network [111]. Similarly, carboxymethyl agarose/chitosan polyelectrolyte complexes formed stable pH-responsive hydrogels with adjustable swelling behavior by varying the ratio of the two polysaccharides. These materials achieved high drug-loading efficiencies and sustained diclofenac release for more than 72 h while maintaining excellent cytocompatibility, demonstrating that electrostatic interactions alone can generate mechanically stable carriers without requiring permanent covalent crosslinking [112].

Several studies have further demonstrated that modifying the polysaccharide architecture rather than introducing new cleavable bonds is sufficient to achieve selective gastrointestinal drug delivery. PEGylated pectin/chitosan nanoparticles designed for oral colon targeting protected phytic acid from premature gastric release, with less than 12% of the encapsulated drug released at pH 1.2 but more than 80% released under intestinal conditions [113]. Likewise, oxidized guar gum crosslinked with chitosan through dynamic Schiff-base interactions produced hydrogels capable of protecting curcumin and aspirin during transit through the upper gastrointestinal tract while enabling controlled release in the colorectum, significantly enhancing the therapeutic potential of combination chemotherapy [114]. These examples highlight that the pH responsiveness of ionizable polysaccharides is particularly advantageous for oral formulations, where predictable physiological pH gradients can be exploited for site-specific delivery.

Natural polysaccharides have also been incorporated into multifunctional responsive systems that combine pH sensitivity with additional environmental triggers. Chitosan and hyaluronic acid derivatives integrated into thermoresponsive poloxamer hydrogels simultaneously improved mechanical stability, pH tolerance, and sustained drug release, yielding cumulative release values exceeding 85% under mildly acidic conditions while preserving excellent cytocompatibility [115]. Similarly, norbornene-functionalized chitosan hydrogels incorporating thermo-responsive poly(N-isopropylacrylamide) crosslinkers exhibited dual pH- and temperature-responsive behavior, allowing minimal release in simulated gastric conditions but nearly complete drug liberation under physiological colonic conditions [116]. These studies demonstrate that ionizable backbone groups can readily be integrated with other responsive mechanisms to achieve higher levels of spatial and temporal control over therapeutic delivery.

Another emerging trend involves combining pH-responsive swelling with dynamic crosslinking strategies that provide additional structural adaptability. Oxidized pullulan derivatives interacting physically or covalently with chitosan generated all-polysaccharide hydrogels whose pore size, swelling behavior, and drug release kinetics could be finely adjusted by selecting aldehyde- or carboxyl-functionalized pullulan derivatives [117]. Likewise, chitosan–maltodextrin nanogels prepared by reductive amination exhibited pH-dependent swelling, increased hydrodynamic diameter under acidic conditions, and excellent colloidal stability, illustrating that nanoscale carriers preserve the same molecular principles governing macroscopic hydrogels [118,119]. These findings emphasize that the physicochemical response of ionizable polysaccharides is maintained across multiple delivery platforms, including hydrogels, nanogels, nanoparticles, and injectable matrices.

Collectively, these studies demonstrate that ionizable backbone groups constitute one of the most versatile molecular strategies for engineering polysaccharide-based drug delivery systems. Unlike chemically cleavable functional groups, these materials regulate therapeutic release through reversible physicochemical changes in polymer conformation driven by protonation–deprotonation equilibria. Consequently, drug release is governed primarily by dynamic modulation of swelling, mesh size, and diffusional transport rather than by degradation of the polymer network. Although this mechanism does not directly generate molecular disassembly or self-reporting signals, it provides a highly biocompatible and predictable approach for controlling release under physiologically relevant pH gradients. This distinction is particularly important within the context of Engineering Release at the Molecular Level, as it illustrates that molecular control of therapeutic delivery may arise either from reversible changes in polymer ionization or from stimulus-induced cleavage of specific functional groups, the latter being addressed in the following sections of this review.

### 5.6. Disulfide, Diselenide and Ditelluride Linkages: Redox-Responsive Polysaccharide-Based Drug Delivery Systems

Disulfide (–S–S–), diselenide (–Se–Se–), and ditelluride (–Te–Te–) linkages provide polysaccharide carriers with covalent junctions that remain comparatively stable under extracellular conditions but cleave in reducing or oxidative pathological environments. Disulfides are predominantly reduced through thiol–disulfide exchange with intracellular glutathione, whereas diselenides respond to both GSH and ROS and generally cleave more readily than sulfur analogues. Ditellurides extend this concept toward highly ROS-sensitive activation. Depending on their position within the carrier, cleavage can detach a drug, disrupt a hydrophobic core, remove a protective shell, or disassemble the entire nanostructure. Chitosan illustrates how both linkage chemistry and surrounding polymer architecture regulate release. Mazzotta et al. linked cysteine and folate to chitosan to obtain folate-targeted nanoparticles that remained stable under physiological conditions but released methotrexate rapidly in a GSH-rich environment, combining receptor-mediated uptake with intracellular reduction [120].

Yuan et al. subsequently showed that increasing the hydrophobic chain length in cystamine-containing chitosan conjugates improved drug loading, cellular uptake, antitumor activity, and redox-triggered doxorubicin release, demonstrating that disulfide cleavage is strongly influenced by hydrophobic core organization [121]. In a complementary design, Zlotnikov et al. combined heparin, chitosan, oleic acid, and lipoic acid to generate pH- and GSH-responsive micelles in which disulfide crosslinks or disulfide-bound doxorubicin increased drug accumulation and selectivity toward tumor cells [122].

Disulfide chemistry has also been applied to polysaccharide prodrugs in which the therapeutic molecule contributes directly to self-assembly. Liu et al. conjugated doxorubicin to dextran–polyethyleneimine through cleavable disulfide spacers, producing 100–140 nm prodrug micelles that released the drug rapidly under reducing conditions, enhanced intracellular accumulation, and improved activity against multidrug-resistant breast cancer cells [123]. Hu et al. developed a hydroxyethyl starch–doxorubicin conjugate that remained stable at extracellular GSH concentrations but released doxorubicin at intracellular levels, resulting in prolonged circulation, enhanced tumor accumulation, improved efficacy, and reduced systemic toxicity [124]. Andrgie et al. similarly prepared an amphiphilic heparin–chlorambucil prodrug containing disulfide bonds; the conjugate self-assembled into vesicular structures and selectively increased toxicity toward tumor cells while remaining compatible with normal cells [125].

Nevertheless, biological targeting can be incorporated through the intrinsic receptor affinity of the polysaccharide. Li et al. used chondroitin sulfate as a CD44-targeting component and conjugated it with a cystamine-modified docetaxel prodrug. The resulting nanoparticles increased docetaxel release under reductive conditions, improved tumor penetration and cytotoxicity, and reduced adverse effects relative to free docetaxel [126]. In other studies, Zhang et al. linked hyaluronic acid and methotrexate through a disulfide bond to produce self-assembled nanoparticles combining CD44 targeting, folate-receptor recognition by methotrexate, rapid redox-triggered disassembly, and improved in vivo antitumor efficacy [127], while Mauro et al. employed an inulin–cysteamine–thiocholesterol conjugate in which redox-sensitive disulfide bridges supported micelle formation and intracellular SN38 delivery, with preferential nuclear payload localization and greater toxicity toward cancer than normal cells [128].

Disulfide linkages also support dual-responsive and route-specific delivery. Liu et al. coupled *Bletilla striata* polysaccharide to stearic acid through cystamine, generating docetaxel-loaded micelles responsive to both acidic pH and reducing conditions and showing enhanced anticancer activity in vitro and in vivo [129]. Cheewatanakornkool et al. used other strategy by prepared oral microbeads containing thiolated pectin–doxorubicin conjugates; cleavage of the disulfide linker under reducing conditions released doxorubicin and changed the release profile from diffusion-controlled behavior toward approximately zero-order kinetics [130].

The use of nanoparticles for oral delivery was also investigated using a crosslinked thiolated sodium alginate with thiolated Eudragit RS100 through disulfide bridges for oral paclitaxel delivery. The nanoparticles increased paclitaxel solubility more than elevenfold, enhanced intestinal permeability 2.19-fold, and produced a relative bioavailability of 237.11% compared with the drug suspension [131].

Another strategy is the replacement of sulfur with selenium leading to broader responsiveness from predominantly reductive cleavage to dual reduction/oxidation sensitivity. Chen et al. connected methoxy-polyethylene glycol to starch through diselenide linkages, producing shell-sheddable micelles disrupted by either GSH or hydrogen peroxide [132]. Their more rapid cleavage relative to disulfides promoted accelerated doxorubicin release in cellular environments. Wang et al. directly compared diselenide- and disulfide-crosslinked carboxymethyl chitosan nanoparticles. Both showed physiological stability, but the diselenide system degraded more rapidly in response to GSH or H_2_O_2_ and achieved greater intracellular doxorubicin accumulation, cytotoxicity, and tumor-growth inhibition [133]. Feng et al. linked hyaluronic acid to chlorin e6 through diselenide bonds, combining CD44-mediated targeting with redox-triggered micellar disassembly and improved photodynamic therapy in CD44-overexpressing breast cancer [134].

Ditelluride chemistry further lowers the oxidative activation threshold. Fan et al. developed ditelluride-bridged carboxymethyl chitosan nanoparticles specifically to respond to the relatively low ROS concentrations encountered in pathological tissues. The system preserved the polysaccharide carrier architecture while enabling highly sensitive ROS-triggered degradation and doxorubicin release [135].

Collectively, these studies show that therapeutic release is governed primarily by the chemical identity and structural position of the redox-sensitive linkage. Disulfides offer established GSH-triggered intracellular activation; diselenides provide faster dual responsiveness to reducing and oxidizing environments; and ditellurides extend sensitivity toward lower ROS levels. The polysaccharide backbone determines properties such as biocompatibility, targeting, mucoadhesion, and circulation, whereas the chalcogen linkage controls when and how the carrier disassembles or liberates its therapeutic cargo.

### 5.7. Azo/Nitro Reduction: Hypoxia-Responsive Functional Groups in Polysaccharide-Based Drug Delivery Systems

Hypoxia-responsive functional groups have emerged as an attractive strategy for engineering tumor-selective drug delivery systems because they exploit one of the most distinctive biochemical characteristics of solid tumors. Rapid cellular proliferation, abnormal vascular architecture, and limited oxygen diffusion generate regions with oxygen tensions below 5 mmHg, whereas healthy tissues typically exhibit oxygen levels around 70 mmHg. This hypoxic microenvironment is accompanied by increased expression of intracellular reductases, including azoreductases, nitroreductases, and NADPH-dependent oxidoreductases, which selectively activate reducible functional groups incorporated into drug carriers. Among the various hypoxia-sensitive moieties, azo (–N=N–) bonds represent one of the most widely investigated molecular triggers due to their high stability under normoxic conditions and rapid enzymatic reduction within oxygen-deficient tissues. Unlike pH- or redox-responsive systems, therapeutic activation is governed by oxygen availability, making azo chemistry particularly attractive for targeting poorly vascularized tumors that are frequently resistant to conventional chemotherapy [136,137].

The molecular mechanism responsible for hypoxia-responsive drug release involves the sequential reduction of the azo bond by intracellular azoreductases using NADPH as an electron donor. Under normoxic conditions, molecular oxygen competes efficiently for electrons, suppressing azo reduction and maintaining carrier stability. In contrast, oxygen deficiency favors electron transfer to the azo linkage, producing unstable hydrazo intermediates that subsequently cleave into aromatic amines. Depending on the molecular architecture, this transformation either disrupts amphiphilic self-assembled nanostructures or disconnects the therapeutic cargo from the polysaccharide backbone, ultimately triggering drug release. Because the reduction process depends directly on oxygen concentration, azo-containing polysaccharides provide highly selective activation within hypoxic tumor regions while minimizing premature drug leakage during systemic circulation [136,137].

Hyaluronic acid has become one of the most extensively investigated polysaccharides for hypoxia-responsive drug delivery owing to its excellent biocompatibility and intrinsic affinity for CD44 receptors overexpressed by numerous malignant tumors. Le et al. synthesized amphiphilic hyaluronic acid conjugates incorporating Black Hole Quencher-3 (BHQ3), an azobenzene derivative containing reducible azo bonds [137]. The conjugates spontaneously self-assembled into nanoparticles capable of encapsulating doxorubicin with high efficiency. Under physiological conditions only approximately 25% of the drug was released after 24 h, whereas hypoxic conditions increased cumulative release to nearly 75%, reflecting rapid cleavage of the azo linker. The nanoparticles also demonstrated enhanced cytotoxicity toward hypoxic 4T1 breast cancer cells together with preferential tumor accumulation in vivo resulting from the combined effects of passive accumulation and CD44-mediated cellular uptake [137].

The same molecular principle has been further expanded toward multifunctional therapeutic systems. Li et al. developed hyaluronic acid-based nanomicelles in which azobenzene simultaneously served as a hypoxia-responsive linker and was combined with esterase-cleavable ester bonds to produce a dual-responsive delivery platform for carmustine. In addition to hypoxia-triggered cleavage of the azo linker, intracellular esterases accelerated micellar destabilization through hydrolysis of ester bonds, providing sequential activation within tumor tissue. Furthermore, the nanocarrier incorporated an O6-benzylguanine analogue capable of inhibiting O6-alkylguanine-DNA alkyltransferase, thereby overcoming an important mechanism of resistance to nitrosourea chemotherapy. The resulting micelles displayed excellent stability during circulation, selective accumulation within tumors, and significantly enhanced antitumor efficacy compared with conventional combination therapy while simultaneously reducing systemic toxicity [138].

Besides conventional chemotherapy, hypoxia-responsive azo chemistry has also been successfully integrated into photodynamic therapy. Lee et al. developed ultra-small hyaluronate dot particles (5–10 nm) incorporating azobenzene linkers together with chlorin e6 (Ce6) [139]. Under normoxic conditions, Ce6 remained in a self-quenched, photodynamically inactive state. Following enzymatic reduction of the azo linkage within hypoxic tumor tissue, cleavage of the linker restored the photoactivity of Ce6, resulting in efficient singlet oxygen generation upon laser irradiation. The combination of excellent tumor penetration resulting from the ultra-small particle size and selective hypoxia-mediated activation significantly enhanced photodynamic efficacy while minimizing phototoxicity toward healthy tissues, illustrating that azo chemistry can regulate not only drug release but also activation of therapeutic function itself [139].

Dextran has likewise proven to be an effective platform for hypoxia-responsive nanocarriers. Son et al. prepared amphiphilic carboxymethyl dextran nanoparticles by conjugating the polymer with BHQ3 containing reducible azobenzene groups [136]. The nanoparticles remained structurally stable under normoxic conditions but underwent rapid dissociation following enzymatic cleavage of the azo linkage in hypoxic cells, producing accelerated intracellular doxorubicin release. Confocal microscopy confirmed oxygen-dependent intracellular drug liberation, whereas biodistribution studies demonstrated preferential accumulation within tumors after systemic administration. These findings established carboxymethyl dextran as an attractive biodegradable carrier capable of combining passive tumor targeting with hypoxia-triggered molecular activation.

Natural polysaccharides have also been exploited to introduce tissue-specific biological functionality beyond passive drug delivery. Liu et al. developed amphiphilic micelles based on Angelica sinensis polysaccharides covalently linked to ferrocene through azobenzene spacers. Besides functioning as a hypoxia-sensitive linker, the azobenzene bond promoted selective release of curcumin under oxygen-deficient conditions, whereas ferrocene catalyzed Fenton reactions that increased intracellular reactive oxygen species and enhanced ferroptosis [140]. The intrinsic affinity of Angelica polysaccharides for hepatocytes further promoted liver targeting through interactions with asialoglycoprotein receptors, illustrating how naturally occurring polysaccharides may simultaneously provide tissue specificity, hypoxia responsiveness, and synergistic therapeutic mechanisms within a single nanoplatform [140].

Although tumor-targeted chemotherapy represents the principal application of hypoxia-responsive polysaccharides, the same molecular principle has also been successfully translated to metabolic diseases. Yu et al. reported a glucose-responsive microneedle patch containing hypoxia-sensitive hyaluronic acid vesicles functionalized with 2-nitroimidazole [141]. Unlike the previous systems, hypoxia was generated locally by glucose oxidase, which consumed oxygen during glucose oxidation. The resulting oxygen depletion reduced nitroimidazole groups to hydrophilic aminoimidazole derivatives, destabilizing the vesicles and rapidly releasing insulin. This strategy provided significantly faster glucose responsiveness than conventional pH-responsive formulations while effectively regulating blood glucose levels in diabetic mice. Although developed for diabetes rather than cancer therapy, this study demonstrates that hypoxia-sensitive functional groups can also exploit artificially generated hypoxic microenvironments, greatly expanding the biomedical applications of reductively cleavable polysaccharide systems [141].

Collectively, azo- and nitro-responsive functional groups demonstrate how enzymatic reduction can be exploited as a highly selective molecular trigger for therapeutic activation. In contrast to pH- or glutathione-responsive systems, activation depends primarily on oxygen availability and reductase activity, providing excellent specificity for hypoxic pathological environments. Incorporation of reducible azo or nitro groups into hyaluronic acid, dextran, Angelica polysaccharides, and other natural polymers has enabled the development of nanocarriers capable of integrating hypoxia-responsive release with receptor-mediated targeting, esterase sensitivity, ferroptosis induction, photodynamic therapy, and glucose-responsive insulin delivery. Within the framework of Engineering Release at the Molecular Level, these systems illustrate how bioreduction of a single functional group can convert an otherwise stable polysaccharide carrier into a selectively activated therapeutic platform whose release profile is dictated directly by the biochemical characteristics of the target tissue.

### 5.8. Ligand-Mediated Targeting Strategies in Polysaccharide-Based Drug Delivery Systems

Although stimuli-responsive drug release significantly improves therapeutic selectivity, the incorporation of targeting ligands into polysaccharide carriers provides an additional level of specificity by promoting receptor-mediated cellular uptake. Natural polysaccharides already exhibit excellent biocompatibility and prolonged circulation; however, conjugation with ligands such as folic acid, biotin, hyaluronic acid, peptides, or carbohydrates enables active recognition of receptors overexpressed on diseased cells. Consequently, active targeting not only increases intracellular drug accumulation but also reduces off-target toxicity and may overcome multidrug resistance by facilitating receptor-mediated endocytosis instead of passive diffusion.

Among the available targeting ligands, folic acid remains one of the most extensively investigated because folate receptors are highly expressed in ovarian, cervical, breast, lung, and head-and-neck cancers while exhibiting limited expression in most healthy tissues. Wang et al. developed a heparin–folate–paclitaxel nanoparticle (HFT-T) capable of selectively targeting folate receptor-positive tumors [142]. Compared with free paclitaxel and non-targeted nanoparticles, HFT-T significantly increased intracellular paclitaxel accumulation and prolonged drug retention within resistant cancer cells. The receptor-mediated uptake partially bypassed P-glycoprotein-mediated drug efflux, resulting in enhanced microtubule stabilization, stronger inhibition of tumor growth, reduced angiogenesis, and improved therapeutic efficacy in multidrug-resistant xenograft models. These findings demonstrate that ligand-mediated internalization can simultaneously improve targeting efficiency and minimize drug resistance.

Biotin has also emerged as an attractive targeting ligand because many rapidly proliferating tumors overexpress sodium-dependent multivitamin transporters that recognize vitamin B7. Deshpande and Jayakannan designed biotin-functionalized dextran vesicles incorporating enzyme- and redox-responsive linkers for targeted doxorubicin delivery [143]. The biotin-modified vesicles displayed strong affinity toward avidin-family receptors, exhibited enhanced uptake by HeLa cervical cancer cells, and produced substantially greater intracellular accumulation than non-targeted systems. Competitive inhibition with free biotin and uptake experiments performed at 4 °C confirmed that receptor-mediated endocytosis represented the dominant internalization pathway. Coupled with intracellular esterase- and glutathione-responsive degradation, the targeted vesicles achieved selective drug release and significantly increased cytotoxicity toward cancer cells while minimizing effects on normal fibroblasts.

Unlike synthetic targeting ligands, hyaluronic acid provides intrinsic biological recognition through its natural affinity for the CD44 receptor, which is overexpressed in numerous solid tumors. This unique characteristic allows hyaluronic acid to function simultaneously as the structural polysaccharide and as the targeting component. Zhang et al. developed hyaluronic acid-coated chitosan nanoparticles carrying paclitaxel prodrugs [144]. The HA shell promoted CD44-mediated endocytosis, while enzymatically cleavable linkages enabled intracellular drug release after cellular internalization. The nanoparticles showed prolonged blood circulation, increased tumor accumulation, efficient reversal of multidrug resistance, and minimal toxicity toward normal fibroblasts. In vivo, the targeted formulation outperformed commercial Taxol by combining active targeting with controlled intracellular activation.

The targeting capability of hyaluronic acid can also be integrated with additional stimuli-responsive mechanisms. Choi et al. reported PEGylated hyaluronic acid nanoparticles in which CD44-mediated uptake was followed by intracellular degradation through hyaluronidase activity. Because hyaluronidase is highly expressed within many tumor tissues, receptor recognition and enzyme-triggered degradation occurred sequentially, allowing efficient intracellular release of the encapsulated drug while maintaining excellent circulation stability before reaching the tumor. This study demonstrated how receptor-mediated targeting and enzyme-responsive activation may function synergistically within a single polysaccharide nanocarrier [145].

Targeting strategies are not limited to cancer therapy. Le-Vinh et al. developed phosphorylated chitosan micelles whose surface charge shifted from negative to nearly neutral after cleavage of phosphate groups by alkaline phosphatase located on epithelial cell surfaces [146]. Although this system does not employ a classical receptor-targeting ligand, it exploits tissue-specific enzyme expression to enhance epithelial interaction after efficient mucus penetration. The dynamic modulation of surface charge substantially improved cellular association while preserving excellent mucus diffusion, illustrating how biological recognition can also be achieved through enzyme-triggered physicochemical transformations.

Similarly, Perera et al. described phosphotyrosine-modified chitosan/carboxymethyl cellulose nanoparticles that undergo alkaline phosphatase-induced inversion of zeta potential from slightly negative to positive values [147]. The initial negative surface favors rapid mucus penetration, whereas enzymatic cleavage exposes cationic groups that promote interaction with negatively charged epithelial membranes. Although originally designed for oral drug delivery, this concept illustrates how biologically responsive surface engineering can complement classical ligand-mediated targeting strategies.

Overall, ligand-mediated targeting has become one of the most effective approaches for enhancing the therapeutic performance of polysaccharide nanocarriers. Folic acid, biotin, and hyaluronic acid represent the most established targeting ligands, each exploiting receptor overexpression to increase selective cellular uptake. When combined with enzyme-, pH-, redox-, or ROS-responsive release mechanisms, these active targeting strategies substantially improve intracellular drug accumulation, reduce systemic toxicity, and help overcome multidrug resistance. The continued integration of receptor-mediated recognition with multiple endogenous stimuli is expected to produce increasingly selective and clinically translatable polysaccharide-based drug delivery systems.

### 5.9. Enzyme-Responsive Peptide Sequences Incorporated into Polysaccharide-Based Drug Delivery Systems

Among the numerous functional groups employed in stimuli-responsive drug delivery, enzyme-cleavable peptide sequences offer the highest degree of biological specificity because therapeutic activation is dictated by the molecular recognition properties of individual proteases rather than by relatively nonspecific physicochemical stimuli such as pH or redox potential. Proteolytic enzymes recognize short peptide motifs with remarkable substrate selectivity, enabling drug carriers to remain stable throughout systemic circulation while undergoing highly localized degradation only after encountering specific disease-associated enzymes. Since many pathological conditions, particularly cancer, are characterized by abnormal expression of proteases involved in extracellular matrix remodeling, lysosomal degradation, apoptosis, or tissue-specific metabolism, incorporation of enzyme-responsive peptide linkers into polysaccharide carriers has become an increasingly attractive strategy for achieving precise spatiotemporal control over therapeutic release. Unlike generic hydrolysable ester or amide bonds, peptide sequences function as molecular recognition elements that encode the biological conditions required for carrier activation, thereby introducing an additional level of programmability into drug delivery systems.

Among all enzyme-responsive peptide motifs, Gly-Phe-Leu-Gly (GFLG) is by far the most extensively investigated sequence because it is efficiently cleaved by cathepsin B, a lysosomal cysteine protease that is markedly overexpressed in numerous solid tumors and metastatic lesions. Following receptor-mediated endocytosis, polysaccharide nanocarriers accumulate within acidic endosomal and lysosomal compartments where cathepsin B hydrolyzes the GFLG sequence, destabilizing the carrier and promoting intracellular drug release. The widespread overexpression of cathepsin B in breast, ovarian, pancreatic, liver, lung, and colorectal cancers has therefore established GFLG as one of the most reliable peptide linkers for tumor-selective intracellular drug activation.

The versatility of GFLG-responsive systems has recently been illustrated by Liu et al., who developed multistage polymeric micelles based on an amphiphilic mPEG–GFLG–RGD–DEVD–doxorubicin conjugate [148]. In this sophisticated architecture, the GFLG sequence served as the first activation step following exposure to lysosomal cathepsin B. Cleavage of GFLG detached the protective PEG corona, exposing the RGD targeting peptide and simultaneously initiating partial drug release. The resulting intracellular apoptosis activated caspase-3, which subsequently cleaved a second peptide sequence (DEVD), producing rapid intracellular release of the remaining doxorubicin. This dual-enzyme cascade significantly enhanced tumor selectivity compared with conventional single-trigger systems, demonstrating that peptide sequences may function not only as degradable linkers but also as sequential molecular switches controlling multiple stages of therapeutic activation [148].

Cathepsin B-responsive GFLG linkers have also been successfully incorporated into polysaccharide-based theranostic systems capable of combining drug delivery with diagnostic imaging. Deshpande et al. reported supramolecular polysaccharide nanotheranostics assembled from β-cyclodextrin derivatives and polysaccharide conjugates carrying both kinase inhibitors and enzyme-activatable fluorescent probes. In this system, proteolytic cleavage by cathepsin B activated fluorescence simultaneously with therapeutic inhibition of intracellular kinase signaling, enabling real-time visualization of treatment response rather than simply monitoring nanoparticle accumulation. Such dual-function platforms illustrate how enzyme-responsive peptide sequences may simultaneously regulate therapeutic activation and generate measurable analytical signals, an important concept for future self-reporting drug delivery systems [149].

Another peptide sequence extensively employed in polysaccharide conjugates is Gly-Gly-Phe-Gly (GGFG), which is likewise recognized by lysosomal cathepsin B. Although differing from GFLG by only one amino acid, GGFG exhibits distinct enzymatic kinetics and has found widespread application in polymer–drug conjugates designed for prolonged systemic circulation. One of the earliest clinically advanced examples is DE-310, a carboxymethyl dextran conjugate of the camptothecin analogue DX-8951f. In this macromolecular prodrug, DX-8951 is covalently attached to carboxymethyl dextran through a GGFG spacer that remains stable during circulation but undergoes efficient proteolytic cleavage after cellular internalization. Detailed structural analysis by Shiose et al. demonstrated that the GGFG-containing conjugates are quantitatively released following enzymatic degradation, confirming the stability and reproducibility of this linker chemistry [150]. Importantly, DE-310 progressed into clinical evaluation, providing one of the earliest demonstrations that enzyme-cleavable peptide sequences incorporated into polysaccharides can achieve successful translational development.

The clinical relevance of GGFG linkers has stimulated the development of increasingly sophisticated dextran-based conjugates. Zhu et al. synthesized maleimide-functionalized dextran capable of conjugating camptothecin derivatives through cathepsin B-sensitive peptide spacers [151]. Following enzymatic cleavage, the active metabolite SN-38 was selectively released while the dextran carrier simultaneously self-assembled into nanoparticles that enhanced passive tumor accumulation through the enhanced permeability and retention (EPR) effect. Compared with free camptothecin analogues, the conjugates exhibited markedly improved aqueous solubility together with sustained enzyme-triggered intracellular activation, illustrating how peptide linkers and polysaccharide carriers may cooperatively optimize both pharmacokinetic and pharmacodynamic properties [152].

More recently, peptide-responsive dextran systems have evolved beyond conventional polymer–drug conjugates toward highly modular therapeutic architectures. Schneider et al. introduced *Dextramabs*, a new class of antibody–drug conjugates in which dextran functions as a multivalent polysaccharide scaffold positioned between trastuzumab and multiple monomethyl auristatin molecules [153]. Although antibody targeting governs tumor recognition, peptide-cleavable linkers regulate intracellular toxin release after lysosomal trafficking. The incorporation of dextran substantially increased drug loading while preserving hydrophilicity and reducing aggregation, overcoming one of the principal limitations of highly loaded antibody–drug conjugates. This work demonstrates that polysaccharides are no longer merely passive carriers but may function as structural platforms enabling precise molecular organization of targeting ligands, peptide linkers, and therapeutic payloads within a single construct.

Besides GFLG and GGFG, other cathepsin-responsive peptide motifs have been explored to further optimize intracellular activation. Short hydrophobic sequences such as ALAL exhibit favorable susceptibility toward lysosomal proteases while providing greater synthetic flexibility during conjugation with polysaccharides. Likewise, the clinically established VC dipeptide, widely employed in antibody–drug conjugates, has recently attracted increasing interest for incorporation into polysaccharide nanocarriers because of its excellent plasma stability and rapid intracellular cleavage. Although reports describing VC-functionalized polysaccharides remain comparatively limited, the success of this sequence in clinically approved conjugates strongly suggests that its integration into biodegradable polysaccharide platforms represents a promising direction for future research.

Collectively, cathepsin-responsive peptide sequences demonstrate how molecular recognition by lysosomal proteases can be exploited to achieve highly selective intracellular drug activation. Whether incorporated into dextran, carboxymethyl dextran, β-cyclodextrin assemblies, or multifunctional polymeric micelles, peptide motifs such as GFLG and GGFG function as programmable molecular switches that remain chemically stable during circulation but undergo rapid enzymatic cleavage following cellular internalization.

Among extracellular proteases, matrix metalloproteinase-2 (MMP-2) has attracted considerable attention because of its central role in tumor invasion, angiogenesis, and extracellular matrix degradation. MMP-2 is highly expressed in numerous solid tumors while remaining comparatively scarce in normal tissues, making it an attractive trigger for extracellular activation before nanoparticle internalization. The peptide sequence PLGLAG has become one of the preferred substrates for MMP-2 owing to its excellent specificity and rapid cleavage kinetics.

An elegant application of this strategy was reported by Han et al., who developed hyaluronic acid end-conjugated poly(amidoamine) dendrimers connected through an MMP-2-sensitive PLGLAG linker [154]. Initially, the nanoparticles possessed diameters of approximately 200 nm, which favored prolonged circulation and efficient tumor accumulation through the enhanced permeability and retention effect. After reaching the tumor microenvironment, cleavage of the PLGLAG sequence by extracellular MMP-2 caused rapid dissociation of the nanoparticles into individual dendrimers of approximately 10 nm. This enzyme-induced size shrinkage dramatically enhanced penetration into dense tumor tissue while preserving the advantages associated with larger nanoparticles during systemic circulation. The study demonstrated that peptide cleavage can regulate not only therapeutic release but also nanoparticle architecture itself, providing a dynamic strategy for overcoming one of the major limitations of cancer nanomedicine [154].

Whereas MMP-2-responsive systems regulate extracellular transport, caspase-responsive peptide sequences exploit intracellular apoptotic signaling to achieve self-amplifying therapeutic activation. Caspase-3, the principal executioner protease of apoptosis, specifically recognizes the tetrapeptide sequence DEVD. Because caspase-3 is activated only after initiation of programmed cell death, DEVD-containing systems establish a unique positive feedback mechanism in which initial drug release induces apoptosis, which subsequently accelerates further drug liberation.

This concept was demonstrated by Liu et al. in their dual-enzyme-responsive polymeric micelles. Following cathepsin B-mediated cleavage of the GFLG sequence and partial release of doxorubicin, intracellular apoptosis activated caspase-3, which then hydrolyzed the DEVD linker to trigger rapid release of the remaining drug payload [148]. Such sequential activation substantially increased intracellular drug concentration while minimizing premature release before apoptosis had been initiated. Compared with conventional single-trigger nanocarriers, the cascade-responsive system exhibited superior selectivity and enhanced antitumor efficacy, illustrating how peptide sequences can be organized into molecular logic circuits rather than functioning as isolated degradable linkers [148].

Another important class of enzyme-responsive peptide motifs exploits prostate-specific antigen (PSA), a serine protease secreted almost exclusively by prostate epithelial cells. Unlike cathepsin B or MMP-2, PSA remains enzymatically active primarily within the extracellular fluid surrounding prostate tumors because circulating serum protease inhibitors rapidly inactivate the enzyme after systemic release. This unique biological compartmentalization provides exceptional disease specificity for prostate cancer.

Owoseni et al. developed a dual-drug conjugate incorporating the peptide sequence HSSKLE, a substrate selectively recognized by PSA. Two independent peptide conjugates carrying SN-38 and cabazitaxel were linked through click chemistry to generate a multifunctional therapeutic platform [155]. Upon exposure to enzymatically active PSA within the prostate tumor microenvironment, selective cleavage of the peptide sequence released both cytotoxic agents while maintaining excellent stability during circulation. The dual-drug conjugate exhibited PSA-dependent cytotoxicity in prostate cancer cells, demonstrating that tissue-specific proteases can regulate combination chemotherapy with remarkable molecular precision [155].

In addition to these well-established peptide motifs, other enzyme-responsive sequences continue to expand the repertoire of programmable polysaccharide carriers. AAN has attracted increasing interest because it is specifically recognized by legumain, an asparaginyl endopeptidase overexpressed in many solid tumors and tumor-associated macrophages. Similarly, alternative cathepsin-sensitive sequences, including ALAL and VC, have demonstrated excellent intracellular stability together with efficient lysosomal cleavage, although their incorporation into polysaccharide systems remains comparatively less explored than GFLG and GGFG. Collectively, these peptide motifs illustrate that small modifications in amino acid composition profoundly influence enzyme specificity, cleavage kinetics, and ultimately therapeutic performance.

Nevertheless, incorporation of imaging probes, fluorescent reporters, or activatable contrast agents into peptide-responsive polysaccharide carriers has enabled the development of theranostic platforms capable of simultaneously monitoring enzymatic activity and therapeutic response. Such systems provide valuable real-time information regarding treatment efficacy and represent an important step toward precision nanomedicine [149].

The remarkable diversity of enzyme-responsive peptide sequences demonstrates that biological recognition can now be programmed with a level of specificity that greatly exceeds that achievable through conventional physicochemical stimuli alone. Whereas pH, redox potential, or ROS concentrations primarily distinguish between normal and pathological tissues, peptide sequences enable discrimination between individual enzymes, cellular compartments, and even specific stages of disease progression. From the clinically advanced GGFG-containing dextran conjugate DE-310 [150] to multistage GFLG/DEVD cascade systems [148], MMP-2-responsive size-shrinkable nanoparticles [154], and PSA-selective dual-drug conjugates [155], peptide engineering has transformed enzyme-responsive drug delivery into one of the most sophisticated strategies currently available for polysaccharide-based nanomedicine.

### 5.10. Glucose-Responsive Drug Delivery

Phenylboronic acid (PBA) has become one of the most extensively investigated molecular receptors for the development of glucose-responsive drug delivery systems because of its unique ability to reversibly recognize cis-diol-containing carbohydrates under physiological conditions. Unlike conventional stimuli-responsive functional groups that rely primarily on bond cleavage, PBA operates through dynamic molecular recognition. The boronic acid moiety reversibly forms cyclic boronate esters with glucose, fructose and other saccharides, establishing an equilibrium whose position depends on glucose concentration, pH and the local chemical environment. This reversible interaction enables continuous modulation of polymer architecture rather than irreversible degradation, providing the molecular basis for self-regulated (“closed-loop”) therapeutic delivery. Consequently, PBA-functionalized polysaccharides have become attractive candidates for diabetes treatment, where insulin release should dynamically follow fluctuations in blood glucose concentration rather than occur according to predetermined release kinetics [156,157].

The molecular mechanism responsible for glucose responsiveness differs fundamentally from the other functional groups discussed in this review. Under physiological conditions, phenylboronic acid exists in equilibrium between neutral trigonal and negatively charged tetrahedral boronate species. Binding of glucose to boronic acid shifts this equilibrium toward the boronate ester, modifying both the hydrophilicity and charge distribution of the polymer. Depending on carrier architecture, this interaction may induce polymer swelling, contraction, network relaxation, dissociation of dynamic crosslinks or competitive displacement of polysaccharide chains from boronic acid binding sites. As a result, therapeutic release can be regulated continuously by glucose concentration without requiring irreversible degradation of the carrier.

Chitosan has become the most extensively investigated polysaccharide for glucose-responsive PBA functionalization because its abundant amino groups facilitate straightforward conjugation while simultaneously lowering the apparent pKa of boronic acid through neighboring amine interactions. One of the earliest demonstrations was reported by Wu et al., who synthesized phenylboronic acid-grafted chitosan nanoparticles capable of encapsulating insulin with excellent biocompatibility and glucose-dependent release [156]. Increasing glucose concentration induced progressive nanoparticle swelling, accelerating insulin diffusion while preserving the structural integrity of the released protein. Circular dichroism analysis confirmed that insulin maintained its native tertiary structure after release, demonstrating that PBA-mediated glucose sensing could be integrated with preservation of protein bioactivity.

Subsequent work focused on improving the selectivity and responsiveness of boronic acid-functionalized chitosan. Siddiqui et al. prepared insulin-loaded chitosan nanoparticles functionalized with 4-formylphenylboronic acid [157]. The nanoparticles exhibited diameters of approximately 140 nm together with insulin encapsulation efficiencies exceeding 80%. Interestingly, insulin release depended not only on glucose concentration but also on the identity of the saccharide. High concentrations of glucose promoted sustained insulin release, whereas fructose produced a different crosslinking behavior owing to its stronger affinity for boronic acid. These findings demonstrated that the dynamic equilibrium between boronic acid and competing diols governs nanoparticle structure and therefore directly regulates therapeutic release.

Because conventional monoboronic acids generally exhibit higher affinity toward fructose than glucose, improving glucose selectivity has become a major objective. Siddiqui et al. addressed this limitation by developing multiboronic acid-conjugated chitosan scaffolds containing multiple boronic acid binding sites [158]. The multivalent architecture substantially improved glucose selectivity within physiologically relevant glucose concentrations while maintaining concentration-dependent insulin release. The study demonstrated that careful molecular engineering of boronic acid density provides an effective strategy for tuning carbohydrate recognition without fundamentally changing the polysaccharide carrier itself.

Besides nanoparticle systems, PBA-functionalized polysaccharides have also been incorporated into mechanically robust glucose-responsive hydrogels. Elshaarani et al. developed chitosan-reinforced hydrogels composed of poly(acrylamide-co-3-acrylamidophenylboronic acid) and chitosan-grafted maleic acid [159]. Unlike conventional glucose-responsive gels that simply swell upon glucose binding, these hydrogels exhibited a characteristic swelling–shrinking transition. At relatively low glucose concentrations, formation of 2:1 boronate-glucose complexes promoted network contraction, whereas higher glucose concentrations favored 1:1 complexation, resulting in hydrogel swelling and accelerated insulin diffusion. Incorporation of chitosan improved both the mechanical properties and glucose responsiveness while simultaneously increasing insulin encapsulation efficiency, illustrating how polysaccharides can enhance the performance of synthetic glucose-responsive networks.

The versatility of phenylboronic acid chemistry extends beyond insulin delivery. Wang et al. exploited the reversible interaction between boronic acid and the keto-enol structure of curcumin to fabricate phenylboronic acid-conjugated chitosan nanoparticles with exceptionally high drug-loading efficiency [160]. Formation of reversible boronate ester interactions stabilized curcumin under physiological conditions while simultaneously allowing pH- and ROS-triggered intracellular release. In addition to hydrogen bonding between chitosan and curcumin, boronate ester formation significantly increased drug loading compared with conventional physical encapsulation methods. The nanoparticles exhibited excellent biocompatibility together with enhanced antitumor activity in both two-dimensional cultures and multicellular tumor spheroids, demonstrating that PBA chemistry is equally valuable for oncology applications beyond glucose-regulated insulin delivery.

More recently, glucose-responsive systems have evolved toward dual-sensing platforms that combine several glucose-responsive mechanisms. Wang et al. constructed polysaccharide nanoparticles by crosslinking phenylboronic acid-modified hyaluronic acid with dextran while simultaneously incorporating glucose oxidase [161]. In this architecture, glucose oxidase converted glucose into gluconic acid and hydrogen peroxide, whereas phenylboronic acid functioned as an independent glucose sensor through reversible boronate ester formation. The dual sensing mechanism significantly enhanced both the response rate and release sensitivity compared with conventional PBA-only systems. Following a single administration, diabetic mice maintained normoglycemic blood glucose levels for approximately 17 h, illustrating the potential of integrating dynamic covalent chemistry with enzymatic amplification to approach physiological glucose regulation.

Dynamic boronic acid recognition has also been integrated into supramolecular assemblies. Zhang et al. developed glucose-responsive nanoclusters assembled through host-guest interactions between phenylboronic acid-modified β-cyclodextrin and adamantane-functionalized polyethylenimine [162]. Besides glucose-triggered insulin release, the supramolecular carrier simultaneously delivered plasmid DNA encoding insulin, enabling combined protein delivery and gene therapy. Elevated glucose concentrations disrupted boronic acid-mediated crosslinking, promoting simultaneous insulin release and intracellular gene expression. This study demonstrated that phenylboronic acid chemistry can regulate not only therapeutic release but also multifunctional drug/gene co-delivery strategies.

Phenylboronic acid has likewise been incorporated into advanced polysaccharide architectures beyond chitosan. Mansour et al. developed glucose-responsive multilayer capsules assembled from phenylboronic acid-modified poly(L-lysine) and alginate using layer-by-layer deposition [163]. Glucose binding altered electrostatic interactions within the multilayer structure, providing reversible capsule permeability without complete structural disintegration. More recently, Oh et al. synthesized injectable succinoglycan hydrogels functionalized with phenylboronic acid and crosslinked through dynamic boronate ester interactions with tannic acid [164]. The resulting hydrogels exhibited excellent injectability, autonomous self-healing, and dual responsiveness toward both glucose and pH. Simultaneous changes in glucose concentration and pH produced synergistic acceleration of tannic acid release, illustrating the growing trend toward integrating glucose sensing with additional endogenous stimuli.

### 5.11. Photoresponsive Functional Groups Incorporated into Polysaccharides for Externally Controlled Drug Delivery

Photo-responsive functional groups provide a unique strategy for controlling drug release because activation is governed by an external stimulus rather than by endogenous biochemical signals. Unlike pH-, enzyme-, or redox-responsive systems, light enables precise spatial and temporal regulation of carrier disruption, allowing therapeutic release to be initiated only at the irradiated site. Most polysaccharide-based photo-responsive systems exploit photocleavable *o*-nitrobenzyl derivatives or coumarin-based chromophores, whose irradiation induces bond cleavage, network degradation, or nanoparticle destabilization without requiring changes in the physiological environment. Consequently, the photoresponsive functional group, rather than the polysaccharide backbone itself, dictates the release mechanism.

Among photocleavable groups, *o*-nitrobenzyl derivatives remain the most extensively investigated owing to their predictable photolysis and compatibility with biological systems. Peng et al. incorporated *o*-nitrobenzyl moieties into dextran-based hydrogels prepared by thiol–Michael addition with dithiolated PEG, generating covalently crosslinked matrices under physiological conditions without radical initiators or catalysts. Upon UV or two-photon near-infrared irradiation, cleavage of the *o*-nitrobenzyl linker induced hydrogel degradation and controlled release of encapsulated green fluorescent protein, demonstrating that photocleavage can simultaneously regulate matrix integrity and protein delivery [165].

The same photochemistry has also been exploited to engineer dynamic three-dimensional cellular microenvironments. Cai et al. combined thiol–Michael hydrogel formation with *o*-nitrobenzyl alcohol photochemistry and matrix metalloproteinase-sensitive crosslinkers to construct hyaluronic acid hydrogels in which biochemical cues could be introduced with microscale spatial precision after gel formation [166]. Rather than serving primarily as drug carriers, these photo-patterned hydrogels demonstrated how light-responsive functional groups can dynamically regulate cell adhesion, migration, and morphogenesis by locally modifying the biochemical composition of the extracellular matrix analogue.

Photo-responsive polysaccharide nanocarriers have likewise been developed to achieve light-triggered nanoparticle disruption. El Founi et al. prepared dextran-coated poly(*o*-nitrobenzyl acrylate) nanoparticles in which the hydrophobic PNBA core underwent photolysis after UV irradiation, leading to destabilization of the nanostructure while preserving excellent colloidal stability before activation [167]. Importantly, dextran provided a biodegradable and biocompatible alternative to PEG as the hydrophilic shell, and the nanoparticles maintained essentially complete Caco-2 cell viability following irradiation, highlighting the potential of polysaccharide-coated photoresponsive carriers for controlled drug delivery.

More complex photo-responsive architectures have subsequently combined photocleavage with covalent shell stabilization. Di Cicco et al. developed multilayer nanocapsules composed entirely of biocompatible materials, including glycol chitosan, heparin, lecithin, and soybean oil [168]. A photoinitiator-free thiol–ene click reaction generated a stable crosslinked shell, whereas an *o*-nitrobenzyl derivative introduced photocleavable junctions that enabled wavelength-controlled release of encapsulated curcumin. The resulting nanocapsules exhibited excellent in vitro biocompatibility and were further validated in a xenograft mouse model, illustrating the feasibility of translating photo-responsive polysaccharide nanocarriers toward in vivo applications.

Alternative photo-responsive chemistries have also been incorporated into polysaccharide carriers. Feng et al. functionalized carboxymethyl chitosan with photocleavable coumarin derivatives to obtain amphiphilic micelles for the controlled release of 2,4-dichlorophenoxyacetic acid [169]. Simulated sunlight cleaved the coumarin groups, altered the hydrophilic–hydrophobic balance of the polymer, destabilized the micelles, and promoted release of the encapsulated compound. Beyond demonstrating efficient light-triggered release, the study highlighted the tunable absorption properties of coumarin chromophores, which permit activation over a broader wavelength range than conventional *o*-nitrobenzyl groups.

Photo-crosslinking strategies have also been employed to simplify the fabrication of polysaccharide nanogels. Lu et al. synthesized *o*-nitrobenzyl alcohol-modified carboxymethyl chitosan that underwent rapid self-photo-crosslinking under 365 nm irradiation without emulsifiers, catalysts, or external crosslinkers [170]. The resulting nanogels exhibited uniform particle size, high doxorubicin loading, excellent physiological stability, and enhanced antitumor activity after lactobionic acid functionalization. Unlike conventional photoresponsive systems, irradiation in this case was used to construct a stable nanogel network rather than to trigger its degradation, illustrating that photoactive functional groups can regulate both carrier fabrication and therapeutic performance.

Photoresponsive functional groups have emerged as powerful molecular tools for regulating drug delivery because they enable precise spatiotemporal control over therapeutic release through external light stimulation. Unlike endogenous stimuli such as pH or redox gradients, light can be applied on demand with high temporal and spatial precision, allowing activation only at the desired site. Current photoresponsive polysaccharide systems mainly exploit three complementary molecular mechanisms: photocleavage of light-sensitive bonds, reversible photoisomerization of azobenzene derivatives, and photocrosslinking reactions that dynamically regulate network architecture. Consequently, the release behavior is governed primarily by the incorporated photoactive functional group rather than by the polysaccharide backbone itself.

Photocrosslinking represents one of the simplest approaches for constructing light-responsive polysaccharide hydrogels. Bukhari et al. developed cinnamoyl-modified alginate hydrogels in which UV irradiation induced initiator-free [2+2] cycloaddition between cinnamoyl groups, generating cyclobutane bridges that increased crosslinking density without requiring external crosslinkers or radical initiators [171]. Drug release could be tuned by both the degree of cinnamoyl functionalization and the irradiation time, demonstrating that photocrosslinking provides a convenient strategy for regulating hydrogel permeability while preserving the intrinsic biocompatibility of alginate.

Azobenzene remains the most extensively investigated photochromic functional group because its reversible trans–cis photoisomerization produces large conformational changes while maintaining chemical integrity. Pang et al. introduced azobenzene side chains onto hyaluronic acid to obtain a water-soluble photoresponsive macromolecule that retained rapid UV-induced isomerization, slow thermal recovery, excellent fatigue resistance over repeated switching cycles, and good cytocompatibility [172]. The use of hyaluronic acid simultaneously improved aqueous solubility and reduced the cytotoxicity typically associated with free azobenzene derivatives, illustrating how polysaccharides can overcome important limitations of conventional photoswitches.

The reversible nature of azobenzene photoisomerization has subsequently been exploited to fabricate dynamic supramolecular assemblies. Pang et al. prepared self-assembled nanoparticles through host–guest interactions between azobenzene-functionalized polymers and β-cyclodextrin-containing polymers [173]. Light irradiation reversibly modified nanoparticle size, regulated drug release, and, after folic acid functionalization, improved targeted delivery toward MCF-7 cells, demonstrating that photoisomerization can simultaneously control nanostructure organization and therapeutic release.

Later, Mena-Giraldo et al. translated this concept into targeted intracellular delivery by grafting azobenzene onto *N*-succinyl chitosan to generate photoresponsive polymeric nanocarriers further functionalized with a cardiac-targeting peptide [174]. Encapsulated model compounds were rapidly released after brief UV irradiation, while targeted cellular uptake increased intracellular cargo accumulation, highlighting the potential of photoresponsive polysaccharide nanocarriers for precise intracellular drug delivery.

Since photoresponsive systems are intended for biomedical applications, biological safety remains a critical requirement. Londoño-Berrío et al. therefore evaluated the cytotoxicity and genotoxicity of azobenzene-based polymeric nanocarriers prepared from biopolymer-functionalized azobenzene derivatives [175]. At concentrations relevant for drug delivery, the nanoparticles exhibited good cellular compatibility, limited DNA damage, and efficient light-triggered release of encapsulated model compounds, supporting the feasibility of azobenzene-based photoresponsive carriers for biomedical use.

Beyond nanoparticle destabilization, azobenzene can also function as a reversible molecular crosslinker. Di Martino et al. synthesized water-soluble cationic azobenzenes capable of crosslinking alginate through supramolecular interactions [176]. The resulting hydrogels underwent rapid and reversible gel–sol transitions upon alternating UV and visible light irradiation, demonstrating that reversible photoisomerization can dynamically regulate hydrogel mechanics without permanent bond cleavage.

More recently, Knaipp et al. incorporated azobenzene directly as a covalent bridge within dextran networks to obtain sustainable photoresponsive polymers and hydrogels [177]. Reversible E/Z photoisomerization altered the mechanical properties of the materials, producing light-induced softening while preserving network integrity. Rather than promoting cargo release through carrier degradation, this strategy illustrates how photoresponsive functional groups can be exploited to modulate the mechanical behavior of polysaccharide biomaterials.

Overall, photo-responsive polysaccharide-based delivery systems demonstrate that light can regulate therapeutic release through several distinct molecular mechanisms, including photocleavage of *o*-nitrobenzyl groups, reversible photoisomerization of azobenzene derivatives, and photocrosslinking of cinnamoyl moieties. Although these approaches differ in their mode of activation, they share a common principle: the release profile is dictated primarily by the chemistry of the photo-responsive functional group rather than by the polysaccharide backbone itself. Polysaccharides such as dextran, alginate, hyaluronic acid, and chitosan mainly provide biocompatibility, biodegradability, and favorable physicochemical properties, whereas the incorporated chromophore determines whether irradiation induces carrier disassembly, reversible structural switching, or network formation. Recent developments further illustrate a transition from simple light-triggered release toward multifunctional systems that integrate photoresponsiveness with targeting ligands, supramolecular assembly, or tunable mechanical properties, expanding the potential of these materials beyond drug delivery to include tissue engineering, dynamic biomaterials, and controlled cell regulation. Consequently, photo-responsive functional groups represent a versatile molecular platform for engineering spatiotemporal control over therapeutic release while preserving the intrinsic advantages of natural polysaccharide carriers.

### 5.12. Ureidopyrimidinone (Upy)-Functionalized Polysaccharides

Ureidopyrimidinone (UPy) has emerged as one of the most effective supramolecular motifs for the functionalization of polysaccharides due to its ability to form highly stable yet reversible quadruple hydrogen bonds. Unlike dynamic covalent systems, UPy-functionalized polymers rely on non-covalent interactions that continuously dissociate and reform under physiological or externally applied stimuli, providing reversible network assembly without the need for chemical crosslinkers. Dextran, cellulose nanocrystals, and cellulose acetate have all been successfully modified with UPy groups to produce injectable or self-assembling biomaterials with tunable mechanical properties and excellent structural recovery [178,179,180].

The reversible UPy dimerization enables dynamic physical crosslinking, allowing hydrogels to undergo shear-thinning during injection followed by rapid recovery of the three-dimensional network after removal of the applied stress. This behavior has been exploited to produce injectable dextran hydrogels capable of rapid self-integration, sustained protein release, and encapsulation of multiple cell populations for tissue regeneration [180]. Similarly, incorporation of UPy-functionalized cellulose nanocrystals into supramolecular polymer matrices markedly improved mechanical strength while preserving reversible network dissociation under light-induced heating, enabling rapid defect repair through temporary disruption and reformation of the hydrogen-bonding network [179]. More recently, cellulose acetate modified with UPy side groups demonstrated that combining biomass-derived polymers with quadruple hydrogen bonding generates materials exhibiting enhanced stretchability, shape-memory behavior, and autonomous self-healing, highlighting the versatility of UPy-mediated supramolecular assembly [178].

From the perspective of stimuli-responsive targeted drug delivery (SRTDD), UPy-functionalized polysaccharides illustrate how reversible supramolecular interactions can regulate material assembly and responsiveness without irreversible chemical transformations. Consequently, UPy-based assemblies represent a valuable intermediate strategy between conventional permanently crosslinked hydrogels and reversible covalent systems based on thermolabile linkages, such as alkoxyamines, where stimulus-induced bond cleavage can be directly coupled with controlled therapeutic release and self-reporting functions.

### 5.13. Diels–Alder Assembly and Retro-Release in Polysaccharide-Based Drug Delivery Systems

Diels–Alder (DA) cycloaddition has emerged as one of the most versatile approaches for engineering polysaccharide-based drug delivery systems owing to its catalyst-free nature, high chemoselectivity, mild reaction conditions, and excellent compatibility with biological molecules. Functionalization of polysaccharides such as hyaluronic acid, chitosan, and carboxymethyl cellulose with furan groups, followed by crosslinking with maleimide-functionalized polymers, enables the formation of injectable hydrogels under physiological conditions while preserving the activity of encapsulated therapeutics [181,182,183]. Compared with conventional irreversible crosslinking strategies, DA reactions generate dynamic covalent networks whose physicochemical properties can be precisely tuned through polymer composition, functionality, and crosslink density.

A defining characteristic of these materials is the reversible nature of the DA cycloadduct. While the forward reaction drives rapid hydrogel formation, the reverse (retro-Diels–Alder) reaction progressively cleaves the crosslinks, leading to network relaxation, increased mesh size, and ultimately controlled release of encapsulated therapeutics. Under physiological conditions, the slow retro-DA reaction contributes to hydrogel degradation and sustained drug release, as demonstrated in hyaluronic acid-based ocular depots capable of releasing bevacizumab for more than 400 days [183]. The retro-DA process can also be externally accelerated by focused ultrasound or mechanical loading, enabling on-demand protein release [184] or force-mediated liberation of small molecules from mechanochemically active double-network hydrogels [185]. These studies highlight that retro-Diels–Alder is not merely a degradation pathway but an effective molecular mechanism for regulating therapeutic release.

Beyond controlled release, DA-mediated crosslinking offers important advantages for biomedical applications. Replacing furan with the more electron-rich methylfuran significantly accelerates gelation at physiological pH, enabling efficient three-dimensional cell encapsulation while maintaining high cell viability [181]. Likewise, systematic rheological studies demonstrated that gelation kinetics, crosslink density, and mechanical properties can be tailored through the concentration and architecture of the reactive groups, whereas elevated temperatures promote retro-Diels–Alder reactions that progressively reduce network stiffness [182]. More recently, injectable carboxymethyl cellulose hydrogels crosslinked through DA chemistry combined rapid in situ gelation with sustained curcumin release and excellent wound-healing performance, further illustrating the translational potential of this reversible covalent assembly strategy [186].

Collectively, these studies demonstrate that reversible DA chemistry provides a robust platform for designing stimuli-responsive polysaccharide delivery systems. The combination of efficient network assembly and programmable retro-release establishes an attractive framework for next-generation self-reporting therapeutic delivery (SRTD) systems. Although current DA-based platforms primarily exploit retro-cycloaddition to regulate drug release, they also illustrate the broader concept that reversible covalent bonds can integrate material assembly with stimulus-responsive disassembly. This principle directly supports the development of thermolabile alkoxyamine (C–ON) linkages, where bond cleavage may simultaneously trigger therapeutic release and generate a measurable analytical signal, representing a key step toward truly self-reporting drug delivery platforms.

## 6. Comparative Overview of Stimuli-Responsive Functional Groups

To facilitate comparison of the stimuli-responsive chemistries discussed above, Table 3 summarizes the activation mechanism, principal advantages, and main limitations of each functional group. No single responsive linkage is optimal for all applications; its selection should balance physiological stability, trigger specificity, cleavage kinetics, biocompatibility, synthetic accessibility, and the characteristics of the intended therapeutic site.

The presented comparison highlights that no single functional group provides an ideal balance between circulation stability, trigger sensitivity, release kinetics, and biocompatibility. Acid-labile, redox-responsive, and enzyme-cleavable linkages exploit biochemical differences between healthy and diseased tissues and therefore enable autonomous drug release. However, their performance may be affected by heterogeneous pH, redox conditions, or enzyme expression, as well as by unintended interactions with components of the systemic circulation.

Photo- and thermo-responsive groups offer greater spatiotemporal control because drug release can be initiated externally. Their clinical applicability is nevertheless constrained by limited light penetration, potential phototoxicity, or the elevated temperatures required to activate certain systems. Strategies based on red-shifted chromophores, near-infrared activation, and thermally responsive groups with lower transition temperatures may help mitigate these limitations, provided that adequate stability under physiological conditions is retained.

The selection of a responsive functional group should be guided by the intended administration route, target-tissue microenvironment, required release profile, carrier architecture, and physicochemical properties of the therapeutic payload. Multi-responsive systems may improve selectivity by integrating two or more biological or externally applied signals, but this added functionality also increases synthetic complexity and may complicate reproducibility, scale-up, and regulatory evaluation. Successful translation will therefore depend on achieving sufficient stimulus selectivity with the simplest and most biocompatible design capable of producing a clinically meaningful therapeutic response.

## 7. Conclusions and Future Perspectives

Natural polysaccharides have evolved from passive carrier materials into versatile molecular platforms for engineering advanced drug delivery systems. Their intrinsic biocompatibility, biodegradability, low immunogenicity, and structural diversity provide a favorable foundation for biomedical applications, but the incorporation of carefully selected functional groups ultimately determines the mechanism, selectivity, and precision of therapeutic release. As discussed throughout this review, dynamic covalent bonds, ionizable moieties, redox-sensitive linkages, photoresponsive chromophores, enzyme-cleavable motifs, and supramolecular recognition elements translate specific endogenous or externally applied stimuli into molecular events such as bond cleavage, reversible association, conformational rearrangement, carrier disassembly, or network swelling.

Despite the remarkable diversity of polysaccharide backbones, common structure–response relationships emerge across virtually all stimuli-responsive systems. Acid-labile groups exploit pathological pH gradients through controlled hydrolysis, boronic acids reversibly recognize cis-diols to support glucose-responsive release, and sulfur-, selenium-, and tellurium-containing linkages respond to reducing or oxidative environments. Photoresponsive chromophores provide externally controlled spatiotemporal activation, whereas enzyme-cleavable motifs exploit disease-associated biochemical activity. These examples collectively demonstrate that the chemical identity, spatial position, and local molecular environment of the functional group govern the activation threshold and release profile, while the polysaccharide backbone primarily provides processability, biological compatibility, targeting potential, and structural support.

A major trend is the transition from single-stimulus carriers toward multifunctional platforms integrating two or more orthogonal activation mechanisms. Combining pH-, redox-, enzyme-, photo-, glucose-, or thermo-responsive functionalities may enable sequential activation as the carrier encounters successive biological barriers, thereby reducing premature leakage and improving the spatial precision of drug release. Advances in supramolecular and dynamic covalent chemistry further introduce reversibility, repeated activation cycles, adaptive behavior, and self-healing properties. However, increasing molecular complexity must be balanced against reproducibility, stability, synthetic accessibility, and manufacturability. Highly sophisticated systems may provide excellent proof-of-concept performance while remaining difficult to standardize or scale.

These developments provide the conceptual basis for the next generation SRTDD systems. Future SRTDD platforms are expected to integrate molecular sensing, therapeutic activation, and feedback within a single functional architecture. In such materials, responsive groups will no longer act solely as passive release switches but as components of adaptive systems capable of adjusting therapeutic output to the evolving biochemical state of a pathological microenvironment. The incorporation of biosensing elements, self-reporting chemistries, and closed-loop regulation could therefore transform polysaccharide carriers into dynamic therapeutic materials that both respond to and report changes in disease-associated signals.

Artificial intelligence and machine-learning approaches may accelerate this transition by supporting the rational selection of polysaccharide backbones, functional groups, crosslinking densities, and substitution degrees. Predictive models trained on molecular descriptors and experimentally measured properties could help relate chemical structure to activation threshold, carrier stability, loading efficiency, mechanical behavior, and release kinetics. Such approaches may reduce empirical trial-and-error experimentation and facilitate the identification of functional-group combinations tailored to specific stimuli or administration routes. Their utility will nevertheless depend on the availability of standardized, high-quality datasets and consistent reporting of material composition, preparation conditions, and performance metrics.

The integration of responsive polysaccharides with precision medicine may further enable carrier design according to patient- or disease-specific biochemical profiles. Variations in enzyme expression, redox state, glucose concentration, pH, and inflammatory activity could be used to select activation chemistries better matched to individual pathological conditions. Personalized drug delivery will therefore require not only adaptable material platforms but also reliable biomarkers and quantitative criteria linking biological stimulus levels to material response. In this context, multifunctional systems should be designed to improve selectivity without creating excessive synthetic or analytical complexity.

Regulatory and industrial considerations will also become increasingly important. Progress toward translation requires reproducible polysaccharide sources, controlled molecular-weight distributions, well-defined degrees of substitution, scalable functionalization procedures, and robust methods for characterizing residual reagents, degradation products, sterilization stability, and long-term storage. Regulatory expectations are likely to favor chemically simple, well-characterized systems in which each functional modification provides a clearly demonstrated benefit. Accordingly, the development of responsive polysaccharide materials should increasingly incorporate quality-by-design principles and standardized structure–property–performance relationships from the earliest stages of material development.

Sustainability should constitute an additional design criterion. Polysaccharides are renewable materials, but their environmental advantage can be diminished by multistep synthesis, toxic solvents, low-yield reactions, or difficult purification. Future strategies should prioritize renewable and traceable sources, aqueous or solvent-minimized processing, catalyst-free or photoinitiator-free reactions, recyclable reagents, and functionalization routes compatible with large-scale manufacturing. Greener chemistry should therefore be considered alongside biological performance rather than treated as a secondary objective.

Ultimately, the future of polysaccharide-based drug delivery lies not only in identifying new carrier materials, but in rationally engineering functional groups that convert chemical, biological, or externally applied information into predictable therapeutic action. The convergence of functional-group chemistry, data-driven materials design, multi-responsive architectures, precision medicine, regulatory-aware development, and sustainable processing is expected to establish a new generation of intelligent polysaccharide biomaterials with improved adaptability, reproducibility, and translational potential.

## Figures and Tables

**Table 1:** Summary of advantages of targeted drug delivery for key therapeutic disciplines.

Therapy Area	Key Advantages of Targeted Delivery
**Oncology**	**Reduced Systemic Toxicity:** Minimizes “off-target” damage to healthy cells (e.g., hair follicles, GI tract), reducing side effects like alopecia and nausea. **Enhanced Permeability and Retention (EPR):** Allows accumulation of drugs specifically within tumor interstitial spaces.
**Antimicrobial Resistance (AMR)**	**Biofilm Penetration:** Carriers can bypass protective bacterial biofilms that standard antibiotics cannot cross. **High Local Concentration:** Delivers a lethal dose directly to the pathogen, reducing the survival of resistant strains.
**Neurological & Neurodegenerative**	**Blood-Brain Barrier (BBB) Crossing:** Enables drugs to bypass the BBB via receptor-mediated transcytosis. **Neuronal Specificity:** Directs treatments to specific cell types (e.g., dopaminergic neurons in Parkinson’s) to slow disease progression.
**Chronic Inflammatory & Autoimmune**	**Site-Specific Immune Suppression:** Targets inflamed joints (Rheumatoid Arthritis) or gut tissue (IBD) without suppressing the entire immune system. **Reduced Steroid Dependence:** Allows for lower systemic doses of potent anti-inflammatories.
**Cardiovascular Diseases**	**Plaque Targeting:** Ligands can target atherosclerotic plaques to deliver thrombolytic or anti-inflammatory agents directly to high-risk areas. **Ischemic Recovery:** Targets drug release to hypoxic (oxygen-deprived) heart tissue following a myocardial infarction.
**Gene Delivery**	**Protection from Degradation:** Prevents enzymes (nucleases) from breaking down DNA/RNA before they reach the target cell. **Intracellular Localization:** Ensures the genetic material reaches the nucleus or cytoplasm specifically where protein expression or silencing is required.

**Table 2:** Comparative properties of the big four polysaccharides used as nanocarriers.

Property	Chitosan	Hyaluronic Acid (HA)	Alginate	Dextran
**Structure**	Linear *β*(1→4) linked *D*-glucosamine.	Linear alternating *D*-glucuronic acid & *N*-acetyl-*D*-glucosamine.	Linear copolymer of *β*-*D*-mannuronic & *α*-*L*-guluronic acid.	Highly branched *α*(1→6) glucose backbone with *α*(1→3) branches.
**Charge**	**Cationic (+)** (at low pH)	**Anionic (−)**	**Anionic (−)**	**Neutral (0)**
**Key Groups**	Primary Amine (-NH_2_), Hydroxyl (-OH)	Carboxyl (-COOH), Hydroxyl (-OH), Amide	Carboxyl (-COOH), Hydroxyl (-OH)	Abundant Hydroxyl (-OH)
**Targeting Context**	**Mucoadhesive:** Sticks to mucosal surfaces (lungs, GI).	**Active Targeting:** Naturally binds to CD44 receptors on cancer cells.	**Site-Specific:** Excellent for oral delivery and intestinal release.	**Stealth:** Low protein adsorption; long circulation in blood.
**Biocompatibility**	High; non-toxic and non-immunogenic.	Excellent; naturally occurring in human ECM.	High; widely used in food and wound dressings.	High; used clinically as a plasma volume expander.
**Biodegradability**	Degraded by **lysozyme** in the body.	Rapidly degraded by **hyaluronidases**.	Partially degradable; often cleared by kidneys if molecular weight is low.	Degraded by **dextranases** (mainly in the colon/liver).
**Common Modifications**	Quaternization (for solubility), Thiolation (for adhesion).	EDC/NHS coupling (to drugs), Methacrylation (for hydrogels).	Ionic crosslinking with Ca^2+^, Oxidation to dialdehyde-alginate.	Esterification (to form micelles), Redox-responsive disulfide linking.

**Table 3:** Summary of key functional group modifications used in stimuli responsive targeted drug delivery.

Functional Group (Trigger)	Mechanism	Key Advantages	Main Limitations
**Hydrazone** pH decrease	Acid-catalyzed hydrolysis of the C=N bond: water addition regenerates the carbonyl compound and hydrazine/hydrazide.	Stable near pH 7.4; cleaves in acidic tumor/endolysosomal compartments; covalent loading limits burst release; reversible bonds can support selfhealing.	Background hydrolysis during circulation; many drugs require derivatization; steric/hydrophobic shielding slows cleavage; potentially toxic hydrazide products.
**Acetal/ketal** pH decrease	Protonation generates an oxocarbenium intermediate; water attack and sequential alcohol loss yield an aldehyde/ketone plus alcohols or a diol.	Low hydrolysis at physiological pH; generally neutral, low-toxicity products; broad polymer placement and tunable cleavage through acetal/ketal structure.	Often slow at mildly acidic extracellular tumor pH; synthesis may require dry, acidic conditions; cleavage depends on water access; aldehydes may react with proteins.
**Orthoester** pH decrease	Stepwise acid-catalyzed hydrolysis via a hemiorthoester produces an ester and alcohols, followed by ester hydrolysis.	Very rapid acid response; poly(orthoester) surface erosion can approximate zero-order release; generally biocompatible products; compatible with hydroxyl-bearing drugs.	Moisture sensitivity and possible premature hydrolysis; demanding synthesis/scale-up; local accumulation of acidic products may irritate tissue.
**cis-Aconityl** pH decrease	Neighboring-carboxyl assistance drives intramolecular attack on the amide, forming a five-membered anhydride and releasing the amine-containing drug.	Sharp cleavage in endolysosomal acidity; accepts many primary-amine drugs; cis-aconitic acid is a biocompatible metabolic intermediate.	Some background hydrolysis; restricted to primary-amine payloads; inactive trans-isomer may form during handling/storage; steric crowding can impede cyclization.
**Boronate ester** pH decrease/glucose	Dynamic B-O ester hydrolysis is accelerated by protonation at low pH; glucose can competitively bind boronic acid and displace the carrier diol.	Reversible, tunable assembly; pKa engineering enables acidic-compartment response; competitive glucose binding supports concentration-dependent, closed-loop release.	Endogenous diols and other sugars reduce selectivity; glucose exchange may be slow; physiological stability often requires electron-withdrawing substituents and added synthesis.
**Disulfide** Redox (GSH)	GSH thiolate attacks sulfur through disulfide exchange, producing mixed disulfides and, after further exchange, thiol-terminated fragments.	Large extracellular-to-intracellular GSH gradient; rapid cytosolic cleavage; versatile for drug conjugation, crosslinking, and carrier disassembly.	Premature reduction by serum thiols or disulfide isomerases; shielding that improves circulation stability also slows GSH access; weak response in low-GSH tumors.
**Diselenide/ditelluride** Redox (GSH/ROS)	GSH induces chalcogen exchange; ROS oxidize Se-Se or Te-Te bonds to chalcogen oxides/acids, weakening and ultimately cleaving the linkage.	Dual GSH/ROS responsiveness; lower activation thresholds and faster cleavage than disulfides; useful in highly oxidative tumors or inflamed tissue.	Potential selenium/tellurium toxicity; more difficult synthesis; light and storage instability, especially for ditellurides; limited organotellurium biocompatibility data.
**Azo** Azoreductase/hypoxia	Enzymatic electron transfer reduces N=N through a hydrazo intermediate; N-N cleavage then forms two primary amine fragments.	Stable in the upper gastrointestinal tract yet cleavable by colonic microbiota; can also target tumor hypoxia; irreversible cleavage prevents re-encapsulation.	Aromatic amine products may be toxic; microbiome-dependent variability; inadequate hypoxia can suppress release; hydrophobic azo carriers may aggregate.
**Nitro** Hypoxia	Reductases convert nitro to nitroso, hydroxylamine, and amine; low oxygen prevents re-oxidation of the initial radical and permits complete reduction.	High hypoxia selectivity; the electron-withdrawing-to-donating and hydrophobic-to-hydrophilic switch can drive self-immolation, swelling, and release; inexpensive building blocks.	Reactive nitroso/hydroxylamine intermediates; requires severe hypoxia and suitable reductases; hydrophobic nitro carriers may have poor solubility.
**Enzyme substrate** Enzyme	Target enzymes catalyze substrate-specific bond cleavage (for example, peptide, ester, or glycosidic linkages), destabilizing the carrier or releasing the payload.	High biochemical specificity; disease-associated enzyme overexpression provides a gradient; catalytic turnover amplifies the release signal.	Bulky enzymes may not reach buried substrates; basal activity in healthy tissue; large interpatient variation in enzyme abundance and activity.
**Azobenzene** Light	Light reversibly switches the elongated trans isomer to the shorter cis isomer; a second wavelength or thermal relaxation restores trans.	Precise, localized, on-demand control; reversible on/off dosing; repeated switching with good photostability.	UV has shallow tissue penetration and can damage DNA; thermal back-isomerization may end the response prematurely; azoreductases can degrade the azo bond.
***o*-Nitrobenzyl** Light	Photoexcitation triggers intramolecular hydrogen transfer and rearrangement; collapse of the intermediate cleaves the benzylic bond and releases acid, alcohol, or amine payloads.	Commercially accessible and easy to incorporate; fast, efficient, irreversible cleavage; broad chemical compatibility.	Usually requires poorly penetrating, phototoxic UV light; o-nitrobenzaldehyde-type products can react with proteins or nucleic acids.
**Coumarin** Light	Excitation weakens the bond at the coumarin alpha-carbon; heterolytic cleavage forms an ion pair, followed by hydrolysis and payload release.	More red-shifted activation than o-nitrobenzyl systems; possible near-infrared two-photon activation; fluorescence enables release tracking; generally biocompatible products.	Spectral tuning can require complex synthesis; lower cleavage quantum yield; hydrophobic carriers may show poor solubility and aggregation.
**Cinnamoyl** Light	UV-induced [2+2] cycloaddition dimerizes adjacent C=C bonds to crosslink the carrier; shorter-wavelength UV cleaves the cyclobutane and releases the payload.	Reversible, stable crosslinked networks; no cleavage byproducts; naturally occurring precursors can be biocompatible; supports reusable on/off release.	Requires high-energy UV with poor penetration and phototoxicity; strict chromophore alignment/proximity; prolonged irradiation can cause irreversible side reactions.
**Ureidopyrimidinone (UPy)** Temperature	Self-complementary quadruple hydrogen-bonded UPy dimers dissociate on heating and reassociate on cooling, reversibly changing carrier crosslink density.	Reversible and byproduct-free; self-healing and shear-thinning behavior enables injectability; modular control of material properties.	Water competes for hydrogen bonds, often requiring hydrophobic shielding; full dissociation may need temperatures above 42–45 °C; synthesis is relatively complex.
**Furan-maleimide** Temperature	Furan and maleimide form a Diels-Alder cycloadduct near ambient/body temperature; heating drives the retro-Diels-Alder reaction and restores the two components.	Water-tolerant dynamic covalent bond; catalyst- and byproduct-free; reversible, repeatable, and self-healing; widely available building blocks.	Retro-Diels-Alder usually requires tissue-damaging temperatures; re-crosslinking is slow at body temperature; maleimides can undergo competing thiol-Michael addition.

## Data Availability

No new data were created or analyzed in this study. Data sharing is not applicable to this article.
